# The primary nitrate response TGA1 and TGA4 transcription factors are negative regulators of sulfate uptake and metabolism

**DOI:** 10.1093/plphys/kiag444

**Published:** 2026-07-06

**Authors:** Suvajit Basu, Varsa Shukla, Ira Trivedi, Pengxin Yu, Hanna Bechtel, Anna Koprivova, Agnieszka Sirko, Anna Wawrzyńska, Daniela Ristova

**Affiliations:** Institute for Plant Sciences, University of Cologne, Zülpicher Str. 47b, Cologne 50674, Germany; Institute for Plant Sciences, University of Cologne, Zülpicher Str. 47b, Cologne 50674, Germany; Institute for Plant Sciences, University of Cologne, Zülpicher Str. 47b, Cologne 50674, Germany; Institute for Plant Sciences, University of Cologne, Zülpicher Str. 47b, Cologne 50674, Germany; Institute for Plant Sciences, University of Cologne, Zülpicher Str. 47b, Cologne 50674, Germany; Institute for Plant Sciences, University of Cologne, Zülpicher Str. 47b, Cologne 50674, Germany; Laboratory of Plant Protein Homeostasis, Institute of Biochemistry and Biophysics Polish Academy of Sciences, Pawińskiego 5a, Warsaw 02-106, Poland; Laboratory of Plant Protein Homeostasis, Institute of Biochemistry and Biophysics Polish Academy of Sciences, Pawińskiego 5a, Warsaw 02-106, Poland; Institute for Plant Sciences, University of Cologne, Zülpicher Str. 47b, Cologne 50674, Germany; Cluster of Excellence on Plant Sciences, Luxemburger Str. 90 (COPT), Cologne 50939, Germany (CEPLAS)

## Abstract

Although the synergistic effects of nitrogen (N) and sulfur (S) on crop performance are well established, the regulatory network integrating these pathways remains largely unknown. Clade I TGA transcription factors (TFs), TGA1 and TGA4, function within the second and third layers of the primary nitrate-responsive transcriptional cascade and are also implicated in immunity.

Here we show that provision of nitrate rapidly induces sulfate uptake and assimilation through a yet undefined mechanism, followed by a delayed repression of *SULTR1;2* mediated by clade I TGA transcription factors. In contrast, clade II TGAs and the nitrate signaling components NRT1.1 and NLP7 were not involved in this regulation. The binding analysis showed that both TGA1 and TGA4 bind to the promoter of the high-affinity sulfate transporter *SULTR1;2*, while in vivo assay confirmed that TGA1 and TGA4 repress the activity of *AtSULTR1;1* and *AtSULTR1;2* promoters. Genetic and biochemical analysis further revealed that clade I TGA TFs negatively regulate sulfur metabolism not only under nitrate provision, but also under sulfate starvation. Accordingly, the *tga1 tga4* double mutant displayed enhanced sulfate uptake, increased cysteine accumulation, and improved fitness under long-term sulfate limitation. Additionally, TGA1 and TGA4 influenced sulfur-containing secondary metabolism, including indolic glucosinolates and camalexin during biotic stress.

By coordinating sulfate acquisition with nitrogen signaling, clade I TGAs contribute to maintaining nutrient homeostasis and plant performance. This work advances our understanding of mineral nutrient integration in plants and provides new perspectives for improving nutrient use efficiency and stress resilience in crops.

## Introduction

Modern agriculture and food production depends on application of fertilizers. Increasing the supply of fertilizers during the Green Revolution resulted in advancement in crop production and enabled feeding the world (Fowler et al. 2013). However, it has also resulted in global environmental repercussions, and current agriculture is faced with an urgent need for sustainable agricultural practices that can improve crop production, while reducing the application of fertilizers (DeLoose et al. 2024). It is estimated that an increase of nitrogen use efficiency (NUE) of only 1% can save more than US$1 billion annually (Kant et al. 2011).

Nitrogen (N) is the key rate-limiting essential nutrient for plant development, and fluctuations in N availability can significantly impact crop growth and yield (Lamig et al. 2022). Nitrate is the major source of inorganic N for plants, and it is taken up by nitrate transporters by the plant roots, such as the transceptor NRT1.1 (NITRATE TRANSPORTER 1.1) (Tsay et al. 1993). Nitrate (NO_3_^−^) is further reduced to nitrite and then ammonium, by nitrate reductase (NR) and nitrite reductase (NIR), respectively (Vidal et al. 2020). However, once nitrate is perceived by NRT1.1 in root cells, a cascade of molecular events occurs, including increased Ca^2+^ concentration, and activation of CPKs (CALCIUM-DEPENDENT PROTEIN KINASEs) kinases that phosphorylate NLP7 (NIN-LIKE PROTEIN 7), another nitrate sensor (Liu et al. 2022) that directly regulates early N-responsive transcription factors (TFs) (Alvarez et al. 2020). NLP7 is the first layer of this nitrate transcriptional cascade, and further layers of TFs response unravel afterwards, including TGA1 and TGA4 (TGACG sequence-specific binding protein 1), HRS1 (HYPERSENSITIVITY TO LOW PI-ELICITED PRIMARY ROOT SHORTENING 1), CDF1 (CYCLING DOF FACTOR 1), LBD37 and LBD38 (LOB DOMAIN-CONTAINING PROTEIN), CRF4 (CYTOKININ RESPONSE FACTOR 4), and others (Lamig et al. 2022). Multiple studies have established a TF hierarchy as a mechanism for transcriptional control and rapid signal propagation within minutes of nitrate provision (Marchive et al. 2013; Varala et al. 2018; Alvarez et al. 2019; Alvarez et al. 2020; Swift et al. 2020). Within this TF hierarchy, TGA1 is positioned as one of the most influential TFs in primary nitrate response (Vidal et al. 2020; Lamig et al. 2022). Together with its homolog TGA4, they regulate many genes that are mostly nitrate-responsive, such as *NRT2.1* and *NRT2.2* (Alvarez et al. 2014). TGA1 also modulates changes in transcriptome in response to increased N concentrations over time. In fact, TGA1 and TGA4 affect gene-regulatory kinetics that proportionally impact plant growth (Swift et al. 2020). *TGA1* and/or *TGA4* expression have been reported to be highly upregulated under drought and osmotic stress (Kilian et al. 2007; van Dijk et al. 2010), but not under sulfate deficiency (Dietzen et al. 2020).

Sulfur (S) is an essential macronutrient in plants, component of proteins, sulfolipids, vitamins, coenzymes, and prosthetic groups (iron–S clusters, lipoic acid, thiamine, coenzyme A, etc) (Ravilious and Jez 2012). The thiol group of cysteine (Cys) forms an intrinsic regulatory mechanism for protein structures via disulfide bonds, while glutathione GSH, and phytochelatins play important roles in mitigating various oxidative stresses and facilitating heavy-metal detoxification (Ravilious and Jez 2012). S plays an important role in the interaction with biotic factors, both symbiotic and infectious bacteria. For instance, the plant growth–promoting bacteria (PGPB) SA187, isolated from desert plants, improves plant stress tolerance by modulating the sulfur metabolic pathway, increasing sulfur as well as cysteine and methionine levels in *Arabidopsis* salt-stressed roots (Andres-Barrao et al. 2021). In the case of pathogenic bacteria, the upregulation of sulfur metabolism was important for pattern-triggered immunity (PTI) (Lovelace et al. 2018). Sulfate (SO_4_^2−^) is the main source of sulfur used by plants. Similarly to nitrate, it is taken up from soil by plant roots by sulfate transporters SULTR1;1 and SULTR1;2 (Yoshimoto et al. 2002). However, SULTR1;2 is the major high-affinity sulfate transporter in Arabidopsis, whereas SULTR1;1 plays a more limited and partially redundant role, with generally weaker phenotypic and transcriptional responses (Shibagaki et al. 2002; Yoshimoto et al. 2002, 2007). Sulfate assimilation occurs mainly in the leaves where the pathway for SO_4_^2−^ reduction to S^2−^ localizes exclusively in the plastids, while Cys biosynthesis takes place in the plastids but also in the mitochondria and the cytosol (Kopriva et al. 2024). In *Arabidopsis thaliana*, sulfate starvation response (SSR) is largely regulated by the key-transcription factor SLIM1/EIL3 (SULFUR LIMITATION 1/ETHYLENE-INSENSITIVE3-like 3) (Maruyama-Nakashita et al. 2006; Dietzen et al. 2020). This includes activation of sulfate acquisition and degradation of secondary S-containing metabolites, glucosinolates (Maruyama-Nakashita et al. 2006; Dietzen et al. 2020). Sulfur deficiency highly upregulates the expression of the 2 sulfate transporters, *SULTR1;1* and *SULTR1;2* (Maruyama-Nakashita et al. 2004), and this upregulation is significantly attenuated in both *slim1* and *eil3* mutant backgrounds (Maruyama-Nakashita et al. 2006; Dietzen et al. 2020; Ristova and Kopriva 2022).

Glucosinolates (GSLs) are class of specialized N- and S-rich metabolites among different species within the Brassicales, including *Arabidopsis thaliana* (Burow and Halkier 2017). GSLs are hydrolyzed upon cell damage by thioglucosidase (myrosinase), and the products are toxic and have protective role against herbivores and pathogens (Burow and Halkier 2017). Under S-deficiency, synthesis of GSLs is suppressed, and the redundant pair of SULFUR DEFICIENCY-INDUCED 1 (SDI1) and SDI2 act as major repressors controlling GSL biosynthesis (Aarabi et al. 2016). GSLs act as nutrient reservoirs for S, since their catabolism is induced under limited sulfate to ensure sulfur recycling back to cysteine (Cys) or back to primary metabolism (Sugiyama et al. 2021). SLIM1/EIL3 plays an important role in the positive regulation of *SDI1* and *BGLU28* (*BETA GLUCOSIDASE 28*) the main enzyme involved in GSLs hydrolysis under S-deficiency (Ristova and Kopriva 2022). N-availability also affects GSLs synthesis proportionally (Jeschke et al. 2019).

The synergistic effect of N and S on crop yield and quality is well known, and the balance of both nutrients is crucial for optimal grain production. Excessive nitrogen application relative to sulfur availability can suppress seed production in rapeseed (Janzen and Bettany 1984). The reciprocal regulatory coupling of responses to N and S limiting conditions on each assimilation pathway is well coordinated, and deficiency of one nutrient represses the other assimilatory pathway (Reuveny et al. 1980). At the transcriptional level, N limitation downregulates expression of genes involved in sulfate assimilation (Yamaguchi et al. 1999; Koprivova et al. 2000; Luo et al. 2020). Reciprocally, S deficiency affects nitrogen transport, assimilation and metabolism, indicating a coordinated regulatory mechanism (Bielecka et al. 2014; Dietzen et al. 2020; Liu et al. 2020). While significant progress has been made in understanding N and S metabolism, the molecular basis of their interconnections, and how one signaling pathway might impinge on the other pathway remains to be discovered.

In this study, we investigated the involvement of the key transcription factors of primary nitrate response, TGA1 and TGA4 (clade I TGAs) in the regulation of sulfate transport and metabolism. We reveal that TGA1 and TGA4 act as negative regulators of sulfate uptake upon nitrate provision, as well as under S-deficiency. The in vitro binding analysis confirmed that TGA1 and TGA4 can bind *SULTR1;2* promoter. Under longer-term S-deficiency the double mutant *tga1 tga4* has increased levels of Cys. While the loss of TGA1 and TGA4 reduces seed yield per plant, sulfate starvation reverses this phenotype and restores fitness. Clade I TGAs additionally negatively regulate Trp-derived secondary metabolites, glucosinolates and camalexin under pathogenic biotic stress (*Burkholderia glumae*).

## Results

### Nitrate provision upregulates sulfate transport and assimilation

**Figure 1:**
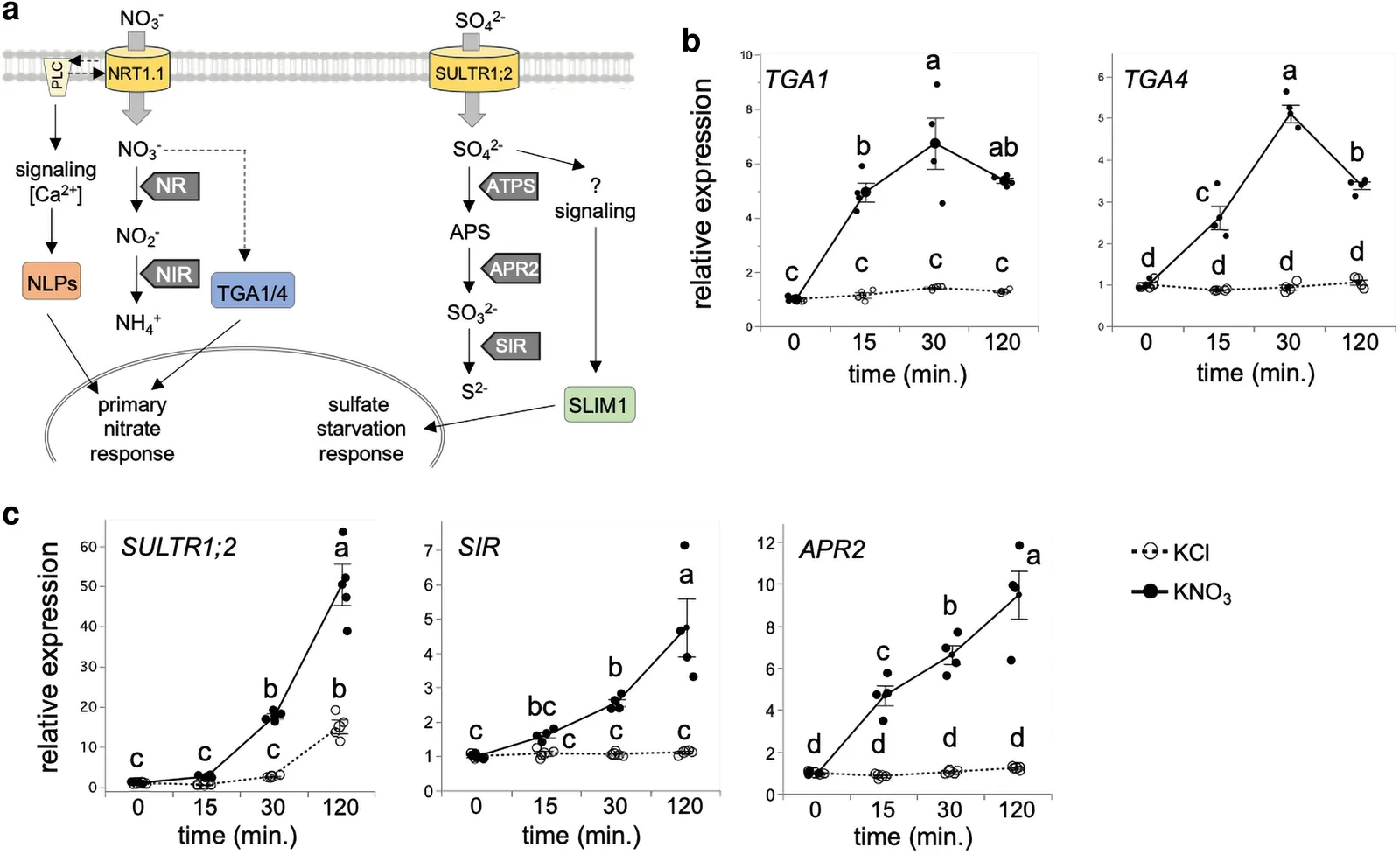
Nitrate provision upregulates expression of sulfate uptake and sulfate assimilation genes. A) Schematic representation of key players in nitrate and sulfate signaling and assimilation. The transceptor NRT1.1 (NITRATE TRANSPORTER 1.1) senses and transports nitrate and further activates PLC (phospholipase C) that triggers Ca^2+^-activated kinases that phosphorylate NLPs (eg NIN-LIKE PROTEIN7) the key-regulator of primary nitrate response (PNR). Nitrate provision also activates another key transcriptional regulator of PNR, the TGA1 and TGA4 (TGACG sequence-specific binding protein 1 and 4). NR (NITRATE REDUCTASE) and NIR (NITRITE REDUCTASE) catalyze the 2 steps of nitrate assimilation. The high-affinity transporter SULTR1;2 (SULFATE TRANSPORTER 1;2) together with SULTR1;1 are taking up sulfate in the roots. The receptor of sulfate starvation remains elusive. Sulfate is further reduced in a few steps catalyzed mainly by ATPS (adenosine-5′-phosphosulfate), APR2 (ADENOSINE-5′-PHOSPHOSULFATE REDUCTASE2), and SIR (SULFITE REDUCTASE). SLIM1 (SULFUR LIMITATION 1) is the central TF that regulates sulfate starvation responses. B) Dynamic response of mRNA for *TGA1* and *TGA4*, and C) *SULTR1;2*, *SIR* and *APR2* transcript levels in wild-type Col-0 (*wt*), in roots of 14-day-old seedlings grown hydroponically, and treated with 1 mM KNO_3_ or 1 mM KCl (as mock treatment) for 15, 30, or 120 minutes. Statistical analysis was performed using two-way ANOVA followed by LS Means *t* test for multiple comparisons for treatment and time, values are means ± s.e.m. (n = 4). Different letters indicate significant differences (*P* < 0.05) between the time and the treatments.

Several comprehensive transcriptomics studies have identified gene regulatory networks responsive to nitrate provision, composed of several dynamic layers of transcriptional factors that regulate multiple points of nitrogen transport, reduction and assimilation (Vidal et al. 2020; Lamig et al. 2022). Among these, genes involved in sulfate transport and assimilation were also regulated by nitrate provision at different timepoints. Comparison of multiple studies revealed many common S-related genes, such as *SULTR1;2*, encoding the main sulfate transporter that has a primary role in sulfate uptake from the rhizosphere (Fig. 1A) (Yoshimoto et al. 2007). Interestingly, significant upregulation of *SULTR1;2* was observed in multiple studies after provision of different concentrations of nitrate. In one study only after 20 minutes of nitrogen provision (60 mM) *SULTR1;2* was upregulated (Varala et al. 2018). In another study *SULTR1;2* was upregulated after 35 minutes of 25 mM nitrate addition (Cheng et al. 2023), while a third study informed about *SULTR1;2* upregulation at a later timepoint of 120 minutes at only 1 mM of nitrate provision (Swift et al. 2020). To confirm the upregulation of the main sulfate transporter upon nitrate provision, we grew wild-type *Arabidopsis thaliana* plants as described in (Swift et al. 2020), and quantified *SULTR1;2* expression at several timepoints after nitrate provision (1 mM) compared to the mock treatment (KCl, 1 mM). To confirm the nitrate provision response, we included known nitrate responsive sentinel gene (Medici et al. 2015; Varala et al. 2018) (Fig. 1B) and also tested upregulation of *TGA1* and *TGA4* (Figure S1), confirming their upregulation at 120 min as previously described (Alvarez et al. 2014). Our results show that nitrate provision triggered rapid and strong upregulation of the 2 sulfate assimilatory genes *APR2* (*ADENOSINE-5′-PHOSPHOSULFATE REDUCTASE2*), at all timepoints tested, while *SIR* (*SULFITE REDUCTASE*) and *SULTR1;2* expression is upregulated later at 30 and 120 minutes (Fig. 1C). Expression of *SULTR1;1* was not significantly different from the mock treatment, and it appears that it does not display nitrate-specific regulation under our conditions, while *SDI1*, an important regulator of sulfur metabolism during sulfur deficiency (Aarabi et al. 2021), was upregulated at 30 and 120 minutes after nitrate provision (Figure S1). These results confirm that sulfate transport and assimilation are dynamically upregulated very early after provision of nitrate to the plant roots.

### TGA1 and TGA4 negatively regulate sulfate transporters, and sulfate uptake

**Figure 2:**
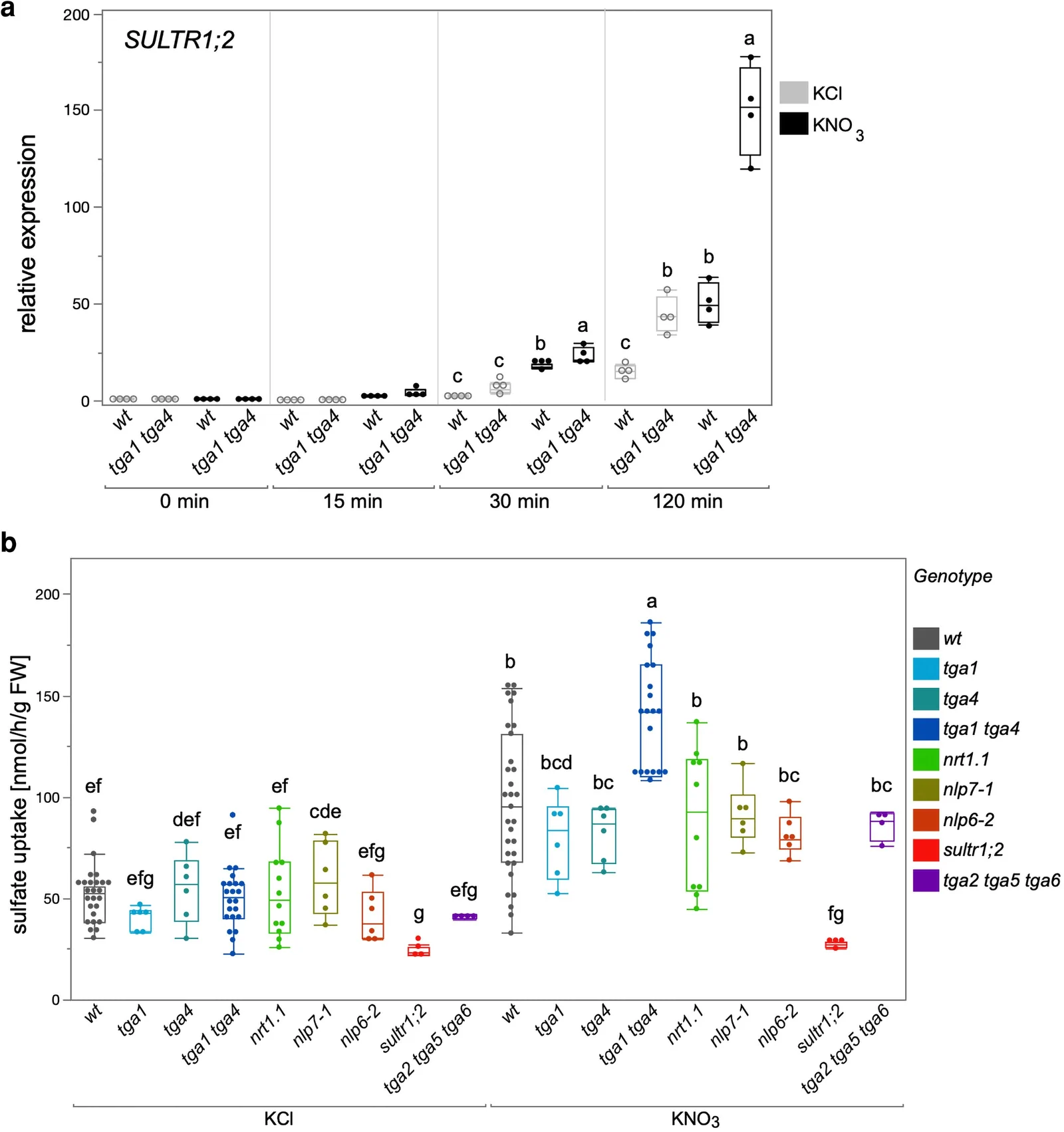
Clade I TGA transcription factors (TGA1 and TGA4) are negative regulators of sulfate transport and sulfate uptake. A) Dynamic response of mRNA for *SULTR1;2* transcript levels in wild-type Col-0 (*wt*), and the double mutant *tga1 tga4* in roots of 14-day-old seedlings grown hydroponically treated with 1 mM KNO_3_ or 1 mM KCl (as mock treatment), for 15, 30, or 120 minutes. Values are means ± s.e.m. (n = 4). Statistical analysis was performed using two-way ANOVA followed by LS Means *t-test* for multiple comparisons for treatment and genotype at each timepoint, values are means ± s.e.m. (n = 4). Different letters indicate significant differences (*P* < 0.05) between the genotype and the treatments. B) Sulfate uptake (µmol/g FW) in *wt*, *tga1, tga4*, *tga1 tga4*, *nrt1.1*, *nlp6-2*, *nlp7-1*, *sultr1;2 and tga2 tga5 tga6* upon nitrate provision at 2 h. Arabidopsis plants (25 seedlings) were grown on a nylon net in hydroculture for 13 days full-media, followed by 24 h of N starvation, and after incubated with control treatment (KCl) or nitrate (KNO_3_), as previously described [Swift et al. 2020]. For sulfate uptake, additional incubation with [^35^S] sulfate for 30 min. was performed, and shoots and roots were harvested separately and extracted with 0.1 M HCl for radioactivity quantification. Statistical analysis was performed using two-way ANOVA followed by LS Means *t* test for multiple comparisons for treatment and genotype, values are means ± s.e.m. (n = 4-20). Different letters indicate significant differences (*P* < 0.05) between the genotypes and the treatments. Detailed *P* values are listed in Table S3.

*SULTR1;2* is ranked among the top genes that are co-expressed with TGA1 and vice versa (Tables S1 and S2). To determine whether the clade I TGAs, TGA1 and TGA4, are involved in the regulation of sulfate transport, we obtained the double mutant *tga1 tga4* (Alvarez et al. 2014) and tested the expression dynamics after nitrate provision. In *tga1 tga4*, upregulation of *SULTR1;2* was 3 times higher at 2 hours after nitrate addition (Fig. 2A). No changes were observed between the wild-type (WT) and *tga1 tga4* mutants for *SIR* and *APR2* (Figure S2).

Next, to verify that the upregulation of *SULTR1;2* impacts sulfate uptake, we quantified total sulfate uptake in 14-day-old seedlings. Since the induction of TGA1 and TGA4 by nitrate was inhibited by 35% to 40% in 2 independent *nrt1.1* mutant backgrounds (Alvarez et al. 2014), we also included the *nrt1.1* mutant (*chl1-5*). Since previous work reported that NLP7 directly regulates *TGA4* (log2FC = 0.8, *P_ad j_* = 2.95^E−08^), but not *TGA1* upon nitrate perception (Alvarez et al. 2020), we also included *nlp7-1* mutant line (Kesarwani et al. 2007). NLP6 does not directly target TGAs, but regulation of some primary nitrate responsive genes is attenuated in the *nlp6-2* mutant (Cheng et al. 2023), thus we also included the *nlp6-2* mutant. TGA TFs in *A. thaliana* are represented by 10 members comprising 5 clades, of which clade I (*TGA1*, *TGA4*) and clade II (*TGA2*, *TGA5*, *TGA6*) are the most studied (Lu et al. 2024). To confirm the specific regulation of clade I TGA TFs, we additionally included the triple mutant of *tga2 tga5 tga6* representing clade II (Yildiz et al. 2023). Finally, we also included the *sultr1;2* mutant line as a control for reduced sulfate uptake (Shibagaki et al. 2002). Our results show that sulfate uptake was induced 2 hours after nitrate addition in all genotypes, except in *sultr1;2*. However, this induction was significantly higher in the *tga1 tga4* (Fig. 2B). We next wanted to test if the higher sulfate uptake in the *tga1 tga4* mutant is specific for nitrate. Thus, we replaced nitrate treatment with ammonium, and quantified sulfate uptake. Ammonium indeed increased sulfate uptake in the WT and the *tga1 tga4* double mutant, but this was not significantly different between the genotypes, suggesting that clade I TGAs regulation on sulfate uptake is specific for nitrate provision, but not ammonium (Figure S3). Taken together, these data show that nitrate provision, induces sulfate transport and uptake, and that clade I TGA TFs act as negative regulators of sulfate transport and uptake, whereas clade II TGAs, and the nitrate sensors NRT1.1, and NLP6, NLP7 are not involved in this regulation.

### TGA1 and TGA4 bind to sulfate transporter promoters

**Figure 3:**
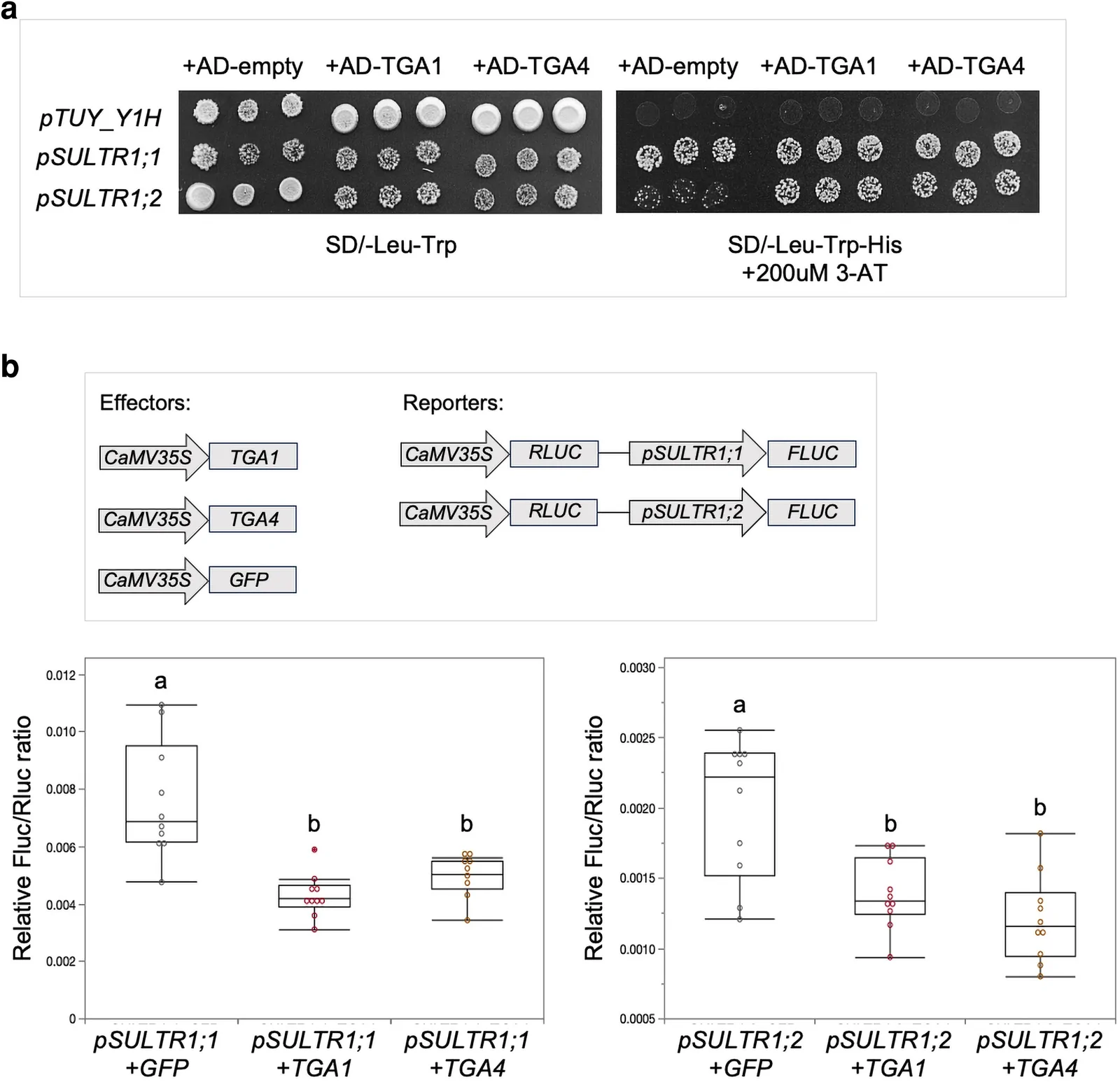
TGA1 and TGA4 binding to the promoters of SULTR1;1 and SULTR1;2. A) Yeast one-hybrid screening showing that TGA1 and TGA4 bind to the *SULTR1;2* promoter. DNA fragments containing promoter sequences of *SULTR1;1*, or *SULTR1;2* were amplified from *A. thaliana* genomic DNA and cloned into pTUY_Y1H vector. The open reading frame of either TGA1 or TGA4 was fused in-frame with the GAL4 activation domain to generate the effector plasmids. Pairs of these reporter and effector plasmids were co-transformed and yeast cells were grown for 7 d at 30 °C on a selective medium lacking leucine (Leu), tryptophan (Trp), and histidine (His) supplemented with 200 mM 3-aminotriazole (3-AT) and photographed. On the left-hand side, yeast growth on the control medium lacking leucine and tryptophan is shown. Three independently transformed yeast colonies are shown. B) Dual luciferase transient transactivation assay in *Nicotiana benthamiana* leaves showing diagram of constructs (upper box). Luciferase (LUC) complementation imaging (LCI) assay showing that AtTGA1 and AtTGA4 repress the *AtSULTR1;1* and *AtSULTR1;2* promoters (lower side). Values are means ± s.e.m. (n = 10). Different letters indicate statistically significant differences (*P* < 0.05) between 2 groups, estimated by one-way ANOVA followed by Tukey's test for multiple comparisons. TGA1 or TGA4 were fused to the C-terminal half of luciferase (cLUC) and *pSULTR1;1* or *pSULTR1;2* were fused to the N-terminal half of luciferase (nLUC). Relative luminescence signal in transfected leaves (n = 9-10), as measured using a CCD camera. As control for transfection efficiency, GFPNLS-GUS was co-expressed in both transfections.

Previous data showed that *SULTR1;2* is one of the direct targets of TGA1 (Swift et al. 2020). To confirm that TGA1 and TGA4 bind to the *SULTR1;2* promoter, we conducted yeast one-hybrid (Y1H) assays and also investigated the binding ability of the TFs to the *SULTR1;1* promoter. TGA binding sites are present in the promoters of *SULTR1;1* and *SULTR1;2* around 400 and 40 bp from transcription start site (TSS), respectively. Yeast strains harboring promoter fragments in combination with an empty vector failed to grow on selective medium, as did strains transformed with the empty pTUY1H vector lacking any promoter insert (Fig. 3A). In contrast, yeast strains co-transformed with the *SULTR1;2* promoter fragment and TGA1 or TGA4 expression plasmids exhibited growth on selective medium, indicating successful protein–DNA interaction. Interestingly, the *SULTR1;1* promoter fragment appeared to autoactivate the reporter gene in yeast, likely due to interaction with endogenous yeast transcription factors (Fig. 3A). Notably, both TGA1 and TGA4 bound with comparable strength to the *SULTR1;2*. Taken together, these findings suggest that *SULTR1;2* is likely a downstream target of TGA1 and TGA4.

### The activity of sulfate transporters is repressed by TGA1 and TGA4

To investigate whether TGA1 and TGA4 transcription factors regulate *SULTR1.1* and *SULTR1.2* promoter activity, we performed dual-luciferase transient transactivation assays in *Nicotiana benthamiana* leaves. Firefly luciferase (FLUC) was driven by the *SULTR1.1* or *SULTR1.2* promoters, while Renilla luciferase (RLUC) under the CaMV35S promoter served as an internal transfection control. Reporter constructs were co-expressed with TGA1, TGA4, or GFP as an effector control. The results showed that co-expression of TGA1 or TGA4 significantly reduced the activities of both *ProSULTR1.1*::FLUC and *ProSULTR1.2*::FLUC compared with the GFP control (Fig. 2B). These findings indicate that TGA1 and TGA4 act as negative regulators of *SULTR1.1* and *SULTR1.2* promoter activity in transient assays, suggesting a repressive role for TGA factors in regulating *SULTR* promoters.

### TGA1 and TGA4 regulate marker genes involved in sulfate starvation response

**Figure 4:**
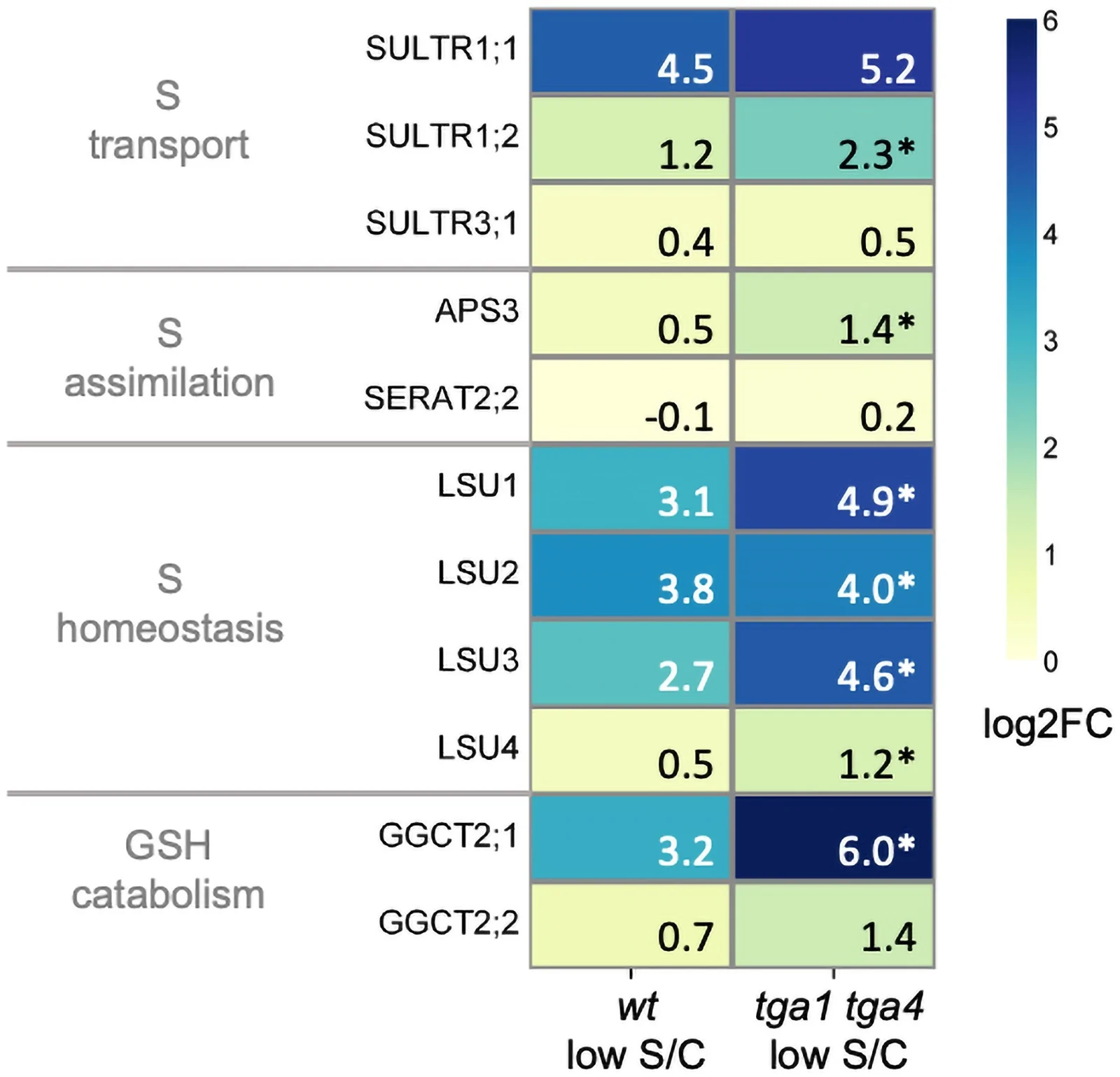
Clade I TGA transcription factors (TGA1 and TGA4) are negative regulators of sulfate transport, assimilation and homeostasis regulation under S-deficiency. Log2 fold-change of *wt* and *tga1 tga4* relative expression response to S-deficiency among eleven related S-metabolism genes. Significantly different means are noted with asterisks. Detailed statistics is listed in Figure S5. Plants were grown vertically on plates for 18 d on either 0.75 mM SO4^2−^ (C) or 0.015 mM SO4^2−^ (low (S) on vertical plates. Relative gene expression was determined via reverse transcription quantitative PCR (RT-qPCR) normalized to two housekeeping genes.

Thirty genes involved in S signaling and metabolism were found to be indirect targets of TGA1 (Swift et al. 2020). To test whether TGA1 and TGA4 regulate response to S deficiency conditions, we grew *A. thaliana* seedlings vertically on plates on full-media and low sulfate (Dietzen et al. 2020), and quantified gene expression in the *tga1 tga4* mutant and WT. We included 2 previously reported direct targets of TGA1 (Swift et al. 2020), *SULTR1;2* and *APS3* (*ATP-SULFURYLASE 3*), as well as indirect targets *GGCT2;1* and *GGCT2;2* (*GAMMA-GLUTAMYL CYCLOTRANSFERASE*) involved in glutathione catabolism and *LSU* genes (*RESPONSE TO LOW SULFUR*), described as hubs for protein-protein interactions under various stresses (Garcia-Molina et al. 2017). We observed significantly higher upregulation of most genes in the *tga1 tga4* mutant than in WT under S starvation condition (Fig. 4 and Figure S4), indicating that TGA1/TGA4 negatively regulate larger set of genes involved in S signaling and metabolism, not only under nitrate provision, but also under longer-term S-deficiency. Taken together, our data strongly suggest a crosstalk of N and S signaling pathways by one of the key TFs of primary nitrate response, the TGA1/TGA4.

### Double mutant *tga1 tga4* exhibits restoration of fitness under S-deficiency

**Figure 5:**
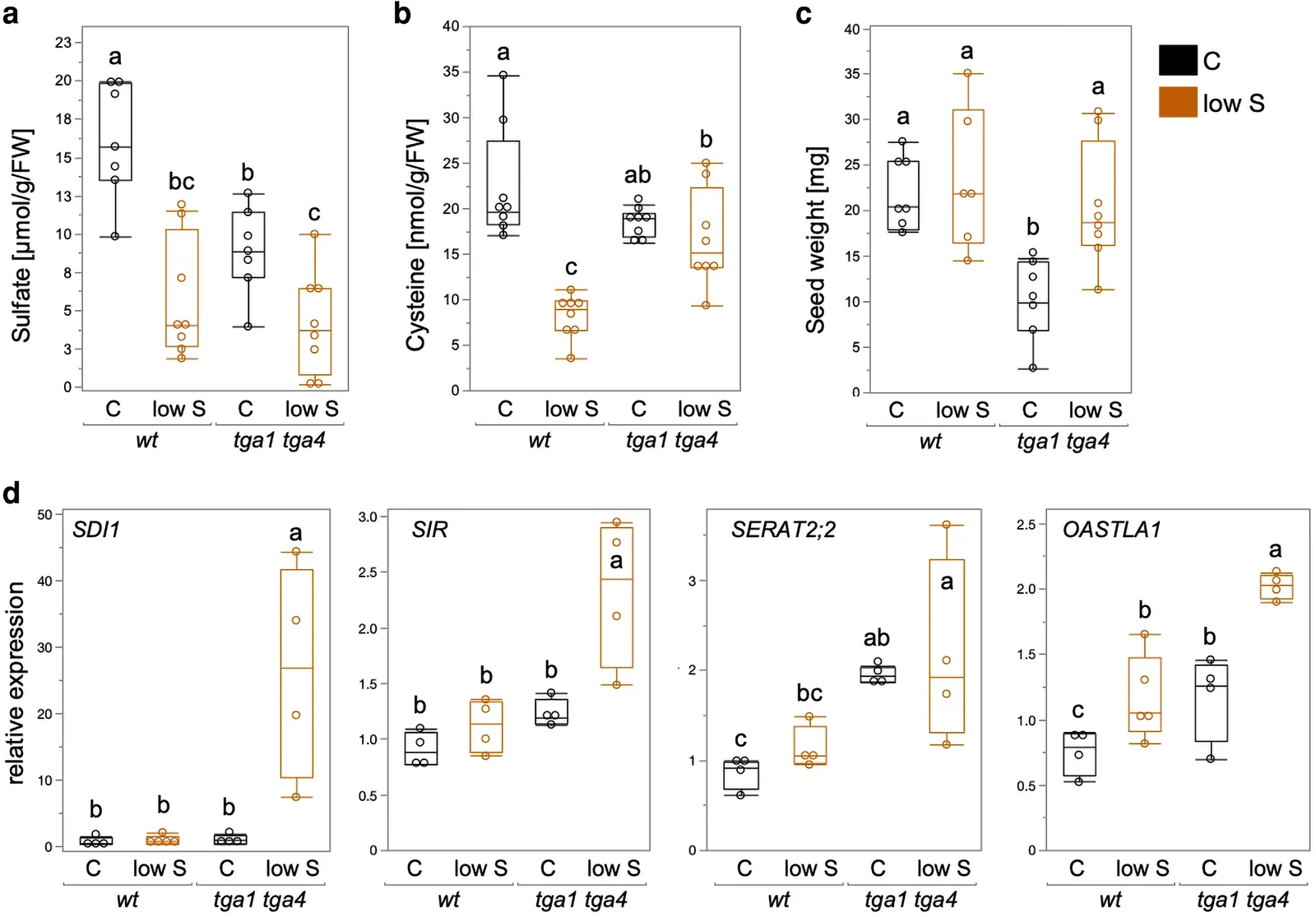
Metabolic and fitness phenotypes in clade I TGA transcription factors (TGA1 and TGA4) under long-term S-deficiency on pots. A) Sulfate anions in shoots (µmol/g FW). B) Cysteine levels (µmol/g FW) in shoots. C) Seed weight per plant (mg). D) Quantitative reverse transcription PCR analysis of *SDI1* and 3 Cys synthesis genes in wild-type Col-0 (*wt*), and *tga1 tga4* shoots, under control (C) and S-deficiency (low S). Wild-type (*wt*), and the double mutant *tga1 tga4* were grown under long-term full-media (C), or S-deficient media (low S) in the greenhouse. Pots with ratio 9:1 (sand:soil) were used and full-media or media lacking sulfate was added twice weekly. For metabolic phenotypes, leaves 7 and 8 were harvested 35 days after sowing, and used for anions, and thiols isolation. Another set of plants were grown to full maturity and fitness (seed weight) was measured. Statistical analysis was performed using two-way ANOVA followed by LS Means *t* test for multiple comparisons for treatment and genotype, values are means ± s.e.m. (n = 4-8). Different letters indicate significant differences (*P* < 0.05) between the genotypes and the treatments. Genes: *SDI1 (SULPHUR DEFICIENCY-INDUCED 1), SIR* (*SULFITE REDUCTASE*), *SERAT2;2* (*SERINE ACETYLTRANSFERASE2;2*), *OASTLA1* (*O-ACETYLSERINE (THIOL) LYASE (OAS-TL) ISOFORM A1*)).

Since we found that TGA1 and TGA4 negatively regulate key sulfate starvation genes, we tested if key S metabolites and general plant fitness are also affected in *tga1 tga4* under long-term S-deficiency. S-deficiency resulted in a significant reduction of sulfate in shoots of 35-day-old WT plants grown in soil, while no difference was observed in the *tga1 tga4* mutant, since it had already decreased sulfate levels under full-media (Fig. 5A). Phosphate levels were the same in both genotypes and conditions, while nitrate levels were higher in the *tga1 tga4* mutant, particularly at low sulfate conditions (Figure S5). Previous data showed that nitrate content in shoots of 7-day-old plants under fluctuating nitrogen condition were reduced, or not different under N deficiency (Kobayashi et al. 2024), suggesting that nitrate content in shoots is dynamically regulated by TGA1/TGA4, environmental fluctuations, such as nitrate and sulfate availability, and developmental period.

Cysteine (Cys) levels were reduced in the WT in response to S-deficiency, while in the *tga1 tga4* Cys levels were not affected (Fig. 5B), suggesting involvement of TGA1/TGA4 in the regulatory mechanisms of cysteine biosynthesis. Glutathione levels were not changed (Figure S4C). To test how the loss of TGA1 and TGA4 affects fitness, we grew WT and *tga1 tga4* plants to full maturity and quantified total seed weight per plant. Sulfate deficiency did not influence seed yield in the WT (Fig. 5C). However, the *tga1 tga4* mutant showed reduced seed weight under full-media, but not under S-deficiency, suggesting restoration of fitness under S-deficiency (Fig. 5C). Next, we tested whether the expression of key genes of Cys synthesis and S homeostasis were altered in the double mutant in the shoots of 35-day-old plants. *SDI1* and genes involved in Cys synthesis showed significant upregulation in the *tga1 tga4* mutant under S-deficiency (Fig. 5D). In summary, the sulfate content and fitness were reduced in the *tga1 tga4* mutant under full-media, but not affected under S deficiency, and Cys content was maintained in the double mutant under S deficiency in line with upregulation of Cys assimilation genes. Thus, these data suggest that increased sulfate transport and uptake in the *tga1 tga4* mutant are important to restore Cys levels and fitness under S-deficiency.

### TGA1 and TGA4 transcription factors negatively regulate glucosinolate biosynthesis under S-deficiency and pathogen infection

**Figure 6:**
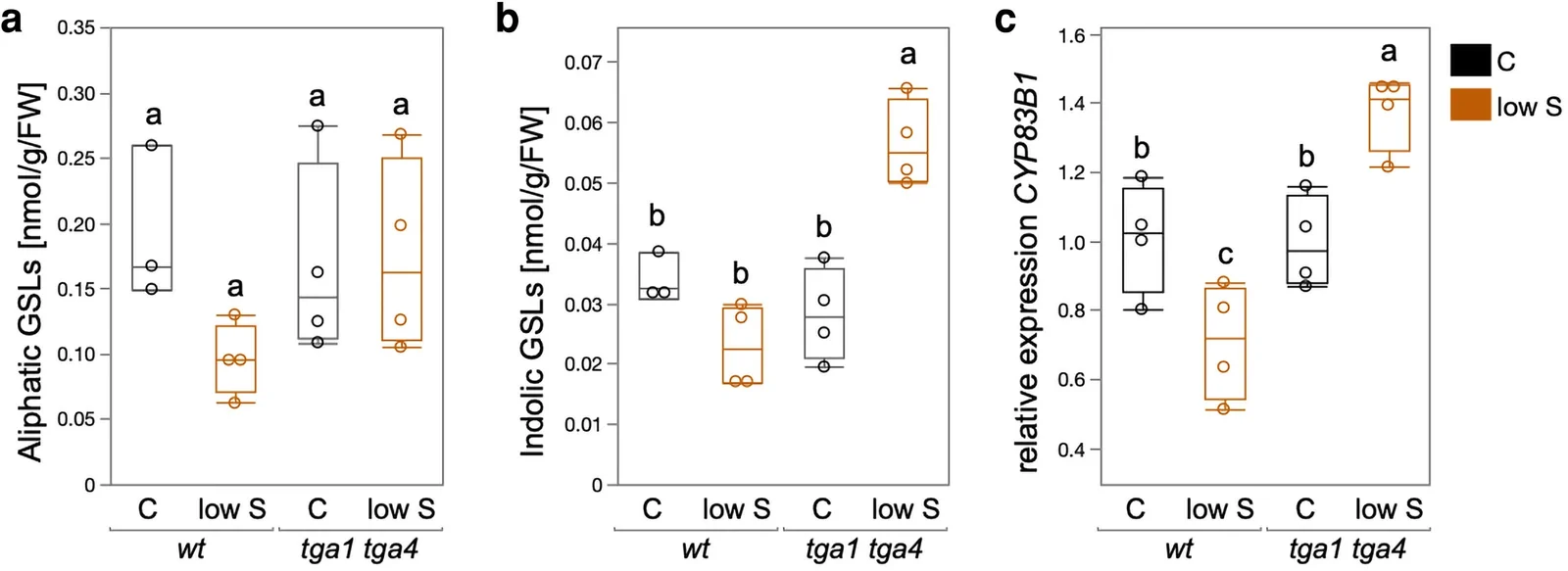
Quantification of glucosinolates and expression *tga1 tga4* mutant infected with BG (*B. glumae* PG1). A) Aliphatic glucosinolates (GSLs) in shoots. B) Indolic glucosinolates (GSLs) in shoots. C) Quantitative reverse transcription PCR analysis of *CYP83B1* that catalyzes the second step of indolic GSLs biosynthesis, in wild-type Col-0 (*wt*), and *tga1 tga4* genotypes. Arabidopsis plants (25 seedlings) were grown on a nylon net in sterile hydroculture for 10 days in 0.75 mM SO4^2−^ (C) or 0.015 mM SO4^2−^ (low S), followed by inoculation with BG or CH bacteria. After 3 days shoots and roots were harvested. Relative gene expression was determined via reverse transcription quantitative PCR (RT-qPCR) normalized to 2 housekeeping genes. Statistical analysis was performed using two-way ANOVA followed by LS Means *t* test for multiple comparisons for treatment and genotype, values are means ± s.e.m. (n = 3-4). Different letters indicate significant differences (*P* < 0.05) between the genotypes and the treatments.

TGA1 and TGA4 are required for full induction by pathogens of *SYSTEMIC ACQUIRED RESISTANCE DEFICIENT 1* (*SARD1*) and *CALMODULIN-BINDING PROTEIN 60g* (*CBP60 g*), that positively regulate salicylic acid (SA) and pipecolic acid (Pip) biosynthesis during systemic acquired resistance (SAR) (Sun et al. 2018). We reanalyzed recent transcriptomic study of *tga1 tga4* plants treated with N-hydroxypipecolic acid (NHP) that accumulates in pathogen-inoculated and distant leaves, and we found that *tga1 tga4* had enriched GO terms of “*sulfur metabolic process*” and “*glucosinolate metabolic process*” (*P* values of 8.64^E−09^, and 1.93^E−08^). Correspondingly, several genes involved in glucosinolate synthesis were upregulated in the *tga1 tga4* mutant in both control and NHP treatment (Yildiz et al. 2023) (Figure S6). Therefore, we sought to test whether glucosinolate synthesis and content were affected by loss of clade I TGA mutants under pathogen infection, and whether sulfate deficiency impacts this regulation. We quantified aliphatic and indolic glucosinolates in 14-day-old plants grown under full or S-deficient media infected with the pathogenic bacteria *Burkholderia glumae* (*BG*) that showed comparable bacterial titer levels in all conditions and genotypes (Figure S6) (Gao et al. 2015; Ross and Somssich 2016; Koprivova et al. 2023). The levels of the aliphatic GSLs were comparable between the 2 conditions and 2 genotypes (Fig. 6A), but the indolic GSLs were significantly higher in the *tga1 tga4* mutant under S-deficiency (Fig. 6B). Specifically, 4-MTB (4-methylthiobutyl), I3M (indol-3-ylmethyl), and 1-MOIM (1-methoxyindol-3-ylmethyl) were significantly higher in the *tga1 tga4* mutant under S-deficiency infected with BG (Figure S8). Expression analysis of few key genes involved in indolic GSLs synthesis showed similar levels with some induction in the *tga1 tga4* mutant under S-deficiency (Figure S9), but *CYP83B1* (*cytochrome P450, family 83, subfamily B, polypeptide 1*) that catalyzes the oxime metabolizing step in indole glucosinolate biosynthesis, was significantly upregulated in the *tga1 tga4* mutant under S-deficiency, similarly to the indolic GSLs (Fig. 6B–6C). Together, these data suggest that TGA1 and TGA4 play a negative role in pathogen-induced indolic GSLs synthesis under sulfate deficiency.

### Clade I TGAs transcription factors negatively regulate camalexin biosynthesis under S-deficiency and pathogen infection

Both indolic GSLs and camalexin are derived from tryptophan, and indole-3-acetaldoxime (IAOx) is the central branching point for multiple Trp-derived secondary metabolites (Mikkelsen et al. 2000). Thus, we next tested whether camalexin synthesis is also affected by loss of TGA1 and TGA4. We infected the WT and the *tga1 tga4* with the pathogenic bacteria *B. glumae* PG1 (BG), known to induce camalexin synthesis (Koprivova et al. 2023). Camalexin accumulation was consistently higher in the *tga1 tga4* mutant when infected with BG (Figure S10). Additionally, a key regulatory gene required for camalexin synthesis *WRKY33* was significantly upregulated in the *tga1 tga4* mutant under pathogenic infection (Figure S10). These results suggest that clade I TGA TFs negatively regulate camalexin under pathogenic bacteria infection.

## Discussion

### Clade I TGA TFs act as a feedback control system of nitrate-induced sulfate uptake

**Figure 7:**
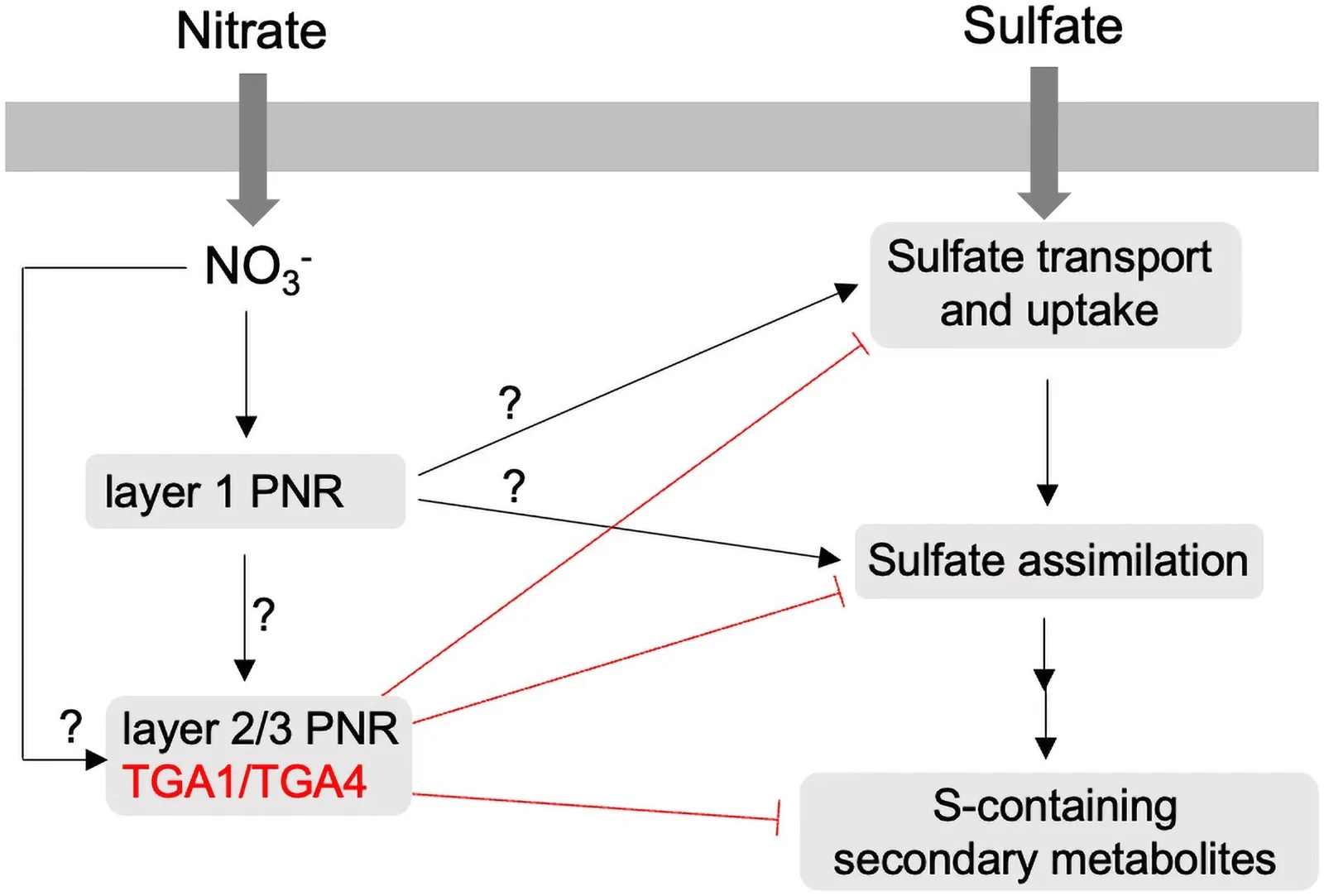
A working model for regulation of sulfate uptake, assimilation and metabolism by the transcription factors TGACG-BINDING FACTOR (TGA1 and TGA4). Upon nitrate provision, sulfate uptake and assimilation are initially increased by an unknown mechanism, possibly by the TFs of the first layer of primary nitrate response (PNR). Later, activation of TGA1 and TGA4, which belong to the second/third layer of PNR, negatively regulate sulfate transport and uptake upon nitrate provision, as well as sulfate assimilation and secondary sulfur metabolites, including Trp-derived indolic glucosinolates (GSLs) and camalexin under sulfate deficiency.

Our results identify clade I TGA transcription factors as a regulatory node linking primary nitrate signaling to sulfur acquisition (Fig. 7) and provide possible mechanistic framework how sulfate uptake can be both rapidly induced and subsequently attenuated following nitrate provision. In WT roots, nitrate rapidly induced key genes of sulfate assimilation within 15-30 min, likely mediated by early transcriptional layer of the primary nitrate response (PNR), while TGA1 and TGA4, belong to the second and third transcriptional layers of the PNR (Lamig et al. 2022). The negative effect of TGA1 and TGA4 on *SULTR1;2* expression is apparent later, suggesting a temporal separation between activation and repression phases. The enhanced *SULTR1;2* expression and sulfate uptake observed in the *tga1 tga4* mutant supports a model in which nitrate signaling initially activates sulfate acquisition to rapidly coordinate sulfur supply with increased nitrogen availability, whereas the delayed action of TGA1/TGA4 establishes a negative feedback loop that subsequently limits the sulfate uptake. Such temporal separation between activation and repression likely enables plants to rapidly respond to nitrogen signals while avoiding excessive sulfate uptake and reduction, processes that are energetically costly and tightly coupled to metabolic demand (Hawkesford 2000; Koprivova and Kopriva 2016).

Our findings further suggest that this repressive branch is specific to clade I TGAs. Sulfate uptake remained unchanged in clade II TGA mutants and in mutants of the nitrate sensor NRT1.1 and the transcriptional regulator NLP7, despite previous evidence that nitrate signaling through NRT1.1 and NLP7 modulates at some degree TGA expression (Alvarez et al. 2014, 2020). This indicates that TGA1/TGA4 likely function downstream or in parallel to canonical nitrate signaling modules and that the partial reduction of TGA expression in the *nrt1.1* mutant is insufficient to relieve repression of sulfate transport. At the same time, nitrate-responsive transcription factors with positive regulatory functions, such as members of the HHO family, have been shown to promote *SULTR1;2* expression (Varala et al. 2018; Brooks et al. 2019). Together, these observations support a model in which nitrate simultaneously activates positive and negative transcriptional arms controlling sulfate uptake: early nitrate-responsive regulators promote sulfate acquisition, whereas the delayed accumulation of TGA1/TGA4 progressively restricts the response. Such antagonistic regulation could provide a mechanism to fine-tune sulfur influx according to assimilation capacity and cellular demand, thereby maintaining nitrogen-sulfur metabolic balance under fluctuating environmental conditions (Jobe et al. 2019). The observation that ammonium stimulates sulfate uptake independently of TGA1/TGA4 further supports the existence of nitrogen source-specific regulatory pathways controlling sulfur acquisition. Together, these findings suggest that clade I TGAs function as a feedback module that integrates nitrate signaling with sulfur metabolism to optimize nutrient homeostasis and minimize unnecessary energetic costs associated with sulfate reduction.

In addition to transcriptional regulation, hormone signaling pathways may contribute to the TGA1/TGA4-mediated attenuation of sulfate uptake. Analysis of publicly available transcriptomic data (Swift et al. 2020) revealed that upon nitrate provision the ABA-responsive transcription factor *ABI5* is specifically reduced in the *tga1 tga4* mutant, whereas it remains unchanged in the wild type (at 120 min.). In parallel, expression of *NCED3*, a key enzyme in ABA biosynthesis, is elevated in *tga1 tga4*, particularly under nitrate supply, suggesting altered ABA homeostasis in the absence of clade I TGAs. Given that ABA is known to influence nutrient uptake and integrate metabolic and stress signals, these observations raise the possibility that TGA1/TGA4 affect sulfate uptake partly through modulation of ABA-related pathways. In contrast, cytokinin-responsive genes such as *ARR5* and *ARR7*, and auxin- responsive genes, such as *IAA19*, *IAA29*, *GH3.3* showed no significant differences between genotypes or treatments, suggesting that cytokinin and auxin signaling are not strongly affected under these conditions and are unlikely to underlie the TGA1/TGA4-dependent regulation of sulfate uptake observed here. We also considered the potential involvement of brassinosteroid signaling considering recent findings implicating BZR1 in the regulation of sulfate uptake (Chen et al. 2025). However, analysis of transcriptomic data (Swift et al. 2020) showed only modest changes in *BZR1* expression upon nitrate provision, with similar responses in both WT and *tga1 tga4* backgrounds, suggesting that brassinosteroid signaling is unlikely to account for the TGA1/TGA4-dependent effects observed in this study. Together, these observations support a model in which nitrate-induced sulfate uptake is fine-tuned not only by direct transcriptional repression via TGA1/TGA4, but also potentially through hormonal pathways, with ABA emerging as a plausible integrative component.

### TGA1 and TGA4 bind to the promoter of SULTR1;2 and repress its activity

Loss of TGA1 and TGA4 caused a significantly amplified nitrate-dependent induction of *SULTR1;2* and a corresponding increase in sulfate uptake, demonstrating that these transcription factors function as negative regulators of sulfur acquisition during nitrate signaling (Fig. 2). This finding was further supported with the Y1H assays confirming that both TGA1 and TGA4 can bind to the *SULTR1;2* promoter, consistent with previous reports identifying *SULTR1;2* as a direct target of TGA1 (Swift et al. 2020). Although binding to the *SULTR1;1* promoter could not be reliably assessed due to autoactivation in yeast, the presence of TGA binding motifs in its promoter suggests potential regulation by these factors. Furthermore, transient dual-luciferase assays demonstrated that co-expression of TGA1 or TGA4 significantly represses the activity of both *SULTR1;1* and *SULTR1;2* promoters in *Nicotiana benthamiana* leaves (Fig. 3). Together these findings indicate that TGA1 and TGA4 interact with sulfate transporter promoters to negatively regulate transcription, revealing a key mechanism by which TGA factors modulate sulfate uptake. Our data indicate that TGA1 and TGA4 act cooperatively in regulating sulfate uptake, as evidenced by the pronounced phenotype observed only in the double mutant (Fig. 2B). While the precise molecular basis of this cooperativity remains to be determined, it likely reflects functional redundancy or coordinated promoter regulation. Further detailed dissection of TGA1/TGA4 interactions at target promoters such as *SULTR1;2* will be an important direction for future studies.

Our findings suggest that the repressive role is specific to clade I TGAs, as sulfate uptake remained unchanged in clade II TGA mutants and in mutants of the nitrate sensor NRT1.1 and the transcriptional regulator NLP7, despite prior evidence that nitrate signaling through NRT1.1 and NLP7 modulates TGA expression (Alvarez et al. 2014, 2020). The persistence of normal sulfate uptake in *nrt1.1* and *nlp7* mutants suggests that TGA1/TGA4 act downstream or in parallel to canonical nitrate signaling modules and that partial reduction of TGA expression in the *nrt1.1* mutant is insufficient to relieve repression of sulfate transport.

While some nitrate-responsive factors promote *SULTR1;2* expression, such as members of the HHO transcription factor family (Varala et al. 2018; Brooks et al. 2019), TGA1 and TGA4 limit the scale of this response. This opposing regulation probably prevents excessive sulfate uptake when the plant cannot efficiently assimilate or use reduced sulfur, thereby avoiding unnecessary energy costs associated with sulfate reduction (Hawkesford 2000; Koprivova and Kopriva 2016), although not directly tested here. Such coordinated control is consistent with broader models of nutrient cross-regulation in plants, in which nitrogen and sulfur pathways are adjusted together to maintain metabolic balance under changing environmental conditions (Takahashi et al. 2011; Jobe et al. 2019). The observation that ammonium stimulates sulfate uptake independently of TGA1/TGA4 further indicates that different nitrogen sources engage distinct regulatory pathways to modulate sulfur acquisition. Together, these findings support a model in which nitrate signaling activates both positive and negative regulatory arms controlling sulfate uptake, thereby ensuring metabolic balance between nitrogen assimilation and the energetically demanding process of sulfate reduction (Hawkesford 2000).

The stronger induction of sulfate starvation marker genes in *tga1 tga4* under sulfur deficiency (Fig. 4) indicates that TGA1/TGA4 normally attenuate starvation signaling. Sulfate deprivation triggers a systemic transcriptional response that enhances transport capacity and reallocates sulfur resources (Nikiforova et al. 2003; Maruyama-Nakashita et al. 2006). Upregulation of this response in the double mutant suggests that TGA1/TGA4 contribute in feedback control mechanisms that fine-tune the amplitude of sulfur deficiency responses. By reducing sulfate starvation signaling, TGA1/TGA4 may help prevent excessive transcriptional reprogramming that could disrupt metabolic homeostasis or divert resources away from growth. Under long-term S-deficiency, WT plants show significant reduction of sulfate, but the *tga1tga4* double mutant has reduced sulfate only under full-media, indicating that TGA1/TGA4 influence long-term sulfur allocation and storage. Cys content was also reduced in the WT in response to S-deficiency, but the maintenance of cysteine levels in the double mutant under S-deficiency, together with increased expression of cysteine biosynthetic genes, suggests compensatory upregulation of assimilatory flux that buffers reduced sulfur pools. Such metabolic compensation is consistent with known regulatory mechanisms that stabilize cysteine levels through coordinated control of sulfate uptake, reduction, and O-acetylserine-dependent signaling (Wirtz and Hell 2006; Hubberten et al. 2012). The reduction in seed weight observed specifically in *tga1 tga4* under control conditions further suggest that deregulated sulfur acquisition and assimilation may sustain a growth cost, potentially due to misallocation of energy and reductant or imbalanced N-S metabolism affecting reproductive development (Hawkesford and De Kok 2006).

The increased accumulation of indolic glucosinolates and upregulation of *CYP83B1* in pathogen-infected *tga1 tga4* plants under S-deficiency suggest that TGA1/TGA4 normally restrict sulfur flux into defense-related secondary metabolism during nutrient limitation. Indolic glucosinolates derive from tryptophan and require substantial reduced sulfur input, linking their synthesis to overall sulfur status (Sonderby et al. 2010). Under sulfur limitation, plants typically prioritize primary metabolism over sulfur-intensive defense compounds (Sonderby et al. 2010). The elevated indolic glucosinolate and camalexin levels in *tga1 tga4* therefore indicate a loss of this prioritization, consistent with a role for TGA1/TGA4 in balancing defense activation with nutrient availability. Given that TGA factors operate within salicylic acid-dependent immune signaling networks, their repressive effect on sulfur allocation to glucosinolate biosynthesis may represent a mechanism that coordinates immune responses with metabolic capacity (Zhang et al. 2003; Sun et al. 2018).

Together, our findings establish clade I TGA transcription factors as key modulators that integrate nitrate signaling with sulfur acquisition, coordinating both primary metabolism and defense metabolites. TGA1/TGA4 attenuate both nitrate-induced sulfate uptake, and sulfur starvation responses, and help balance sulfur allocation between assimilation, storage, and secondary metabolism. Loss of this negative regulation leads to amplified sulfate transport, compensatory maintenance of reduced sulfur pools, and alternation of defense compounds under limitation, with associated growth costs. These results support a model in which TGA1/TGA4 help plants integrate N–S metabolic coupling and aligns immune investment with nutrient status.

## Materials and methods

### Plant material and growth conditions

*Arabidopsis thaliana* Col-0 was used as wild type in all experiments. The double mutant *tga1 tga4* was kindly supplied by Prof. Rodrigo A. Gutiérrez (Santiago, Chile). The triple mutant *tga2/5/6* was kindly supplied by Prof. Jürgen Zeier (Düsseldorf, Germany). The single mutants of *nrt1.1* and *sultr1;2* were previously published (Shibagaki et al. 2002; Guo et al. 2003). T-DNA line for *NPL7* gene was obtained from NASC (SALK_114886), and a homozygous line was isolated for this study. Primers used for genotyping are listed in Table S1.

The seeds were surface sterilized with chlorine gas using 125 ml NaClO and 2.5 ml HCl (37%) for 3 hours under the desiccator; after venting in the sterile hood, sterile H_2_O was added and they were kept 3 days under dark at 4 °C. The seeds were placed onto 0.8% agarose plates containing a modified Long-Ashton medium (Dietzen et al. 2020), or mesh when grown hydroponically, or directly on the soil:sand mix in pots. The media consisted of 1.5 mM Ca(NO_3_)_2_*4H_2_O, 1 mM KNO_3_, 0.75 mM KH_2_PO_4_, 0.75 mM MgSO_4_*7H_2_0, and 0.1 mM Fe-EDTA in terms of macroelements. Microelements consisted of 10 µM MnCl_2_*4H_2_0, 50 µM H_3_BO_3_, 1.75 µM ZnCl_2_, 0.5 µM CuCl_2_, 0.8 µM Na_2_MoO_4_, 1 µM KI, and 0.1 µM CoCl_2_*6H_2_0. In the S-deficient media, the 0.75 mM MgSO_4_*7H_2_0 was replaced with a mixture of 0.7125 mM MgCl_2_×6H_2_O and 0.000225 mM MgSO_4_×7H_2_0. All media were supplemented with 0.8 g/L MES salts, and 0.5% sucrose, and pH adjusted to 5.7 with KOH. The plates were incubated vertically for 18 days (plates), or horizontally for 14 days (hydroponic cultures) in Sanyo light chambers with a photoperiod consisting of a 16:8-h light and dark cycle, with humidity at 60% and 21 °C.

The plants in soil:sand mix in pots were grown to full maturity in the greenhouse. The genotypes were cultivated in growth chambers under long day conditions using 90% autoclaved sand/soil mix under 2 different sulfate concentrations: Control (75 mM SO_4_^2−^), and S-def (0 mM SO_4_^2−^). The experimental design employed randomized complete block design order with 8 replicates per genotype. They were watered with VE water for 2 weeks after sowing seeds and in the beginning of the third week were fertilized according to the respective conditions. At the fifth week, 2 healthy and fully-grown leaves (7th and 8th leaves) were sampled for metabolic analysis, including anion content (nitrate, sulfate, and phosphate) and thiols (cysteine and glutathione), as well as RNA extraction for qPCR. Second set was grown to full maturity and total seed weight was measured per plant.

### Sulfate uptake

For determination of sulfate uptake, the plants were grown in 12-well plates as described in Koprivova et al., (2023). Sterile seeds were distributed onto square sterile nylon membranes and placed in 12-well plates on top of 1 mL of the modified Long-Ashton medium with 0.5% sucrose. After stratification for 2 days in dark and cold, the plates were incubated for 3 days in the dark and in 22 °C to promote etiolation, and further for 2 weeks in the growth cabinets under the long day conditions as described above. For uptake measurement, the medium was exchanged with 1 mL of the Long-Ashton medium with 0.2 mM sulfate supplemented with 12 µCi of [^35^S] sulfuric acid and incubated for 30 min in the light. Whole seedlings still on the mesh were washed thoroughly, blotted dry, and shoot and root samples were cut, weighed, and stored separately in liquid nitrogen until further processing on the same day. Samples were extracted in a 10-fold volume of 0.1 M HCl. One hundred microliters of extract were used to determine sulfate uptake by scintillation counting (Dietzen et al. 2020).

### RNA isolation and expression analysis

Total RNA was extracted from the roots of 18-day-old plants (plates) or 14-day-old plants (hydroponically) by standard phenol/chloroform extraction and LiCl precipitation. Thereafter, DNase treatment was performed, and cDNA was synthesized from 600 ng of total RNA using the QuantiTect Reverse Transcription kit (QIAGEN) according to the manufacturer's protocol. The product was diluted with autoclaved water to a final volume of 200 μL. We used quantitative real-time PCR (qPCR), and gene-specific primers indicated in Table S4. The qPCR was performed using the goTaq→ qPCR Master Mix kit along with SYBR green as per the manufacturer's instructions using a CFX96 Touch Real-Time PCR Detection System (Bio-Rad). All quantifications were normalized to the actin and clathrin genes (Medici et al. 2015) using the 2^−ΔΔCt^ method. Primers used for qPCR are listed in Table S1.

### Anions analysis

For the measurement of phosphate, nitrate, and sulfate anions, 1 mL of sterile H_2_O was added to approximately 20 mg of homogenized shoot material and shaken for 1 h at 4 °C, then heated to 95 °C for 15 min. The samples were centrifuged at maximum speed for 15 min at 4 °C, and 200 μL of the supernatants were transferred to an ion chromatography vial. Standard curves were generated using 0.5, 1, and 2 mM KH_2_PO_4_, KNO_3_, and K_2_SO_4_. The inorganic anions were measured with the Dionex ICS-1100 chromatography system and separated using a Dionex IonPac AS22 RFIC 43 250 mm analytic column (Thermo Scientific). The running buffer was made up of 4.5 mM NaCO_3_ and 1.4 mM NaHCO_3_ as described in Dietzen et al. 2020.

### Thiols analysis

To analyze low molecular weight thiols (cysteine and GSH), approximately 20 mg of homogenized plant material was extracted with 0.1 M HCl at a 1:10 ratio (w/V) and subsequently centrifuged at maximum speed at 4 °C. To reduce the thiols in the samples, 60 μL of the supernatant was transferred to a new tube and 100 μL 0.25 M CHES-NaOH (pH 9.4) was added. Thereafter, 35 μL 10 mM dithiothreitol was added, the tubes were vortexed and incubated for 40 min at room temperature (RT). Five μL of 25 mM monobromobimane was added to the reduced extracts, the samples were vortexed and incubated in darkness for 15 min at RT. The reaction was stopped by adding 110 μL of 100 mM methansulfonic acid and vortexing. After centrifugation at 4 °C for 20 min, 200 μL of supernatant was transferred into high-performance liquid chromatography (HPLC) vials. Standards, ranging from 0 to 100 mM, were prepared using 2 mM L-cysteine and GSH stocks. The conjugated thiols were resolved using reverse phase (RP)-HPLC (Eurospher 100-3 C18, 150 × 4 mm; Knauer) and a gradient of 90% [v/v] methanol and 0.25% [v/v] acetic acid, pH 4.1 in 10% [v/v] methanol and 0.25% [v/v] acetic acid, pH 4.1, and detected fluorimetrically with a 474 detector with an excitation wavelength at 380 nm and emission wavelength at 470 nm. The flow rate was constant at 1 mL min^−1^.

### Glucosinolates analysis

Glucosinolates (GSLs) were extracted from approximately 20 mg homogenized plant material using 500 mL hot 70% (v/v) methanol, and 10 μL of sinigrin was added as internal standard. The extract was vortexed and incubated at 70 °C for 45 min, vortexing twice during the incubation. The samples were left to cool and centrifuged at maximum speed for 5 min at RT. The supernatant was transferred to prepared columns containing 0.5 mL DEAE Sephadex A-25, washed twice with 0.5 mL sterile H_2_O, and subsequently twice again with 0.5 mL of 0.02 M sodium acetate buffer. With a new tube placed underneath each column, a layer of 75 μL of sulfatase was placed onto the column. The samples were left at RT overnight, and the resulting desulfo-GSLs were eluted twice with 0.5 mL sterile H_2_O, followed by a final elution by 0.25 mL. The eluates were combined, vortexed, centrifuged for 5 min, and 200 μL of the supernatant were transferred to HPLC vials. The desulfo-GSLs were resolved by HPLC (Spherisorb ODS2, 250 3 4.6 mm, 5 μm; Waters) using a gradient of acetonitrile in water and detected by UV absorption at 229 nm. The GSLs were quantified using the internal standard and response factors as described in Dietzen et al., (2020).

### Camalexin

Camalexin was extracted in 200 µL from approximately 50 mg leaves as described in (Koprivova et al. 2019). After centrifugation at RT for 20 min, 20 µL supernatants were injected into a Thermo Scientific Dionex UltiMate 3000 HPLC system with Waters Spherisorb ODS-2 column (250 mm × 4.6 mm, 5 µm). The samples were resolved using a gradient of acetonitrile in 0.01% (v/v) formic acid. Camalexin was detected by fluorescence detector with an excitation at 318 nm and emission at 368 nm. For the quantification, external standards were used ranging from 1 pg to 1 ng per µL (Koprivova et al. 2019).

### Co-cultivation with BG

Plants were grown in the 12-well plates as described above for 14 days. For inoculation, overnight bacterial cultures were washed 2 times with sterile 10 mM MgCl2 and final OD600 was measured. The *Burkholderia glumae* (BG) PG1 was diluted stepwise to OD600 = 0.0005 in 10 mM MgCl_2_. Into each well, 8 µL of these suspensions were used for inoculation; 8 µL of 10 mM MgCl_2_ was used as mock treatment. Samples for camalexin (shoots) and DNA (roots) were harvested after 3 days of inoculation.

### Determination of bacterial titer

For the estimation of bacterial titer using qPCR, the method from Ross and Somssich (2016) was adapted, that showed comparable levels in the tested conditions and genotypes (Figure S7). Genomic DNA was extracted using a buffer containing 0.025 M EDTA, 0.2 M Tris (pH 8.0), 0.25 M NaCl, and 0.5% SDS. After 10 min incubation at 65 °C and subsequent centrifugation, the supernatant was precipitated with an equal volume of isopropanol, washed with 70% ethanol and resuspended in 100 µL of sterile water. For the qPCR, 13 ng of corresponding DNA samples were used with Arabidopsis (At primer AT4G26410) and *B. glumae* PG1 specific primers (Burk1 for NR042931). The qPCR conditions were the same as for the expression analysis. The qPCR reactions were performed in duplicate for each of the 4 independent samples. To relate the qPCR results to the bacterial titer, first serial dilutions of bacterial suspensions of different OD_600_ were plated on LB plates and the colonies were counted manually to link OD_600_ and colony forming units (cfu). Subsequently, 10 µL of five 10-fold dilutions of bacterial suspensions with an initial OD_600_ = 1.8 were added to 30 mg of Arabidopsis leaves and the DNA extracted and analyzed as above. Using calibration curves plotting ΔCt (Ct_Bg_–Ct_At_) and the cfu against the log_10_ OD_600_, the bacterial titer can be estimated from the ΔCt values.

### Yeast one-hybrid screening

To construct the reporter plasmid for a yeast one-hybrid (Y1H) assay, DNA fragments containing promoter sequences of *SULTR1;1*, and *SULTR 1;2* were amplified from *A. thaliana* genomic DNA and cloned into MCS of pTUY1H vector (Castrillo et al. 2011) on the *Sac*I and *Mlu*I restriction sites. The open reading frame of either TGA1 or TGA4 was fused in-frame with the GAL4 activation domain in a pDEST22 (Invitrogen) to generate the effector plasmids. Pairs of these reporter and effector plasmids were introduced into yeast strain Y187 (Takara Bio USA) and the transformants were selected on SD me­dium lacking leucine and tryptophan. Transformed colonies were then spotted on SD–His/−Leu/–Trp medium with 200 mM 3-aminotriazole and cultured at 30 °C for 7 days. All plasmids used in the assay are described in Table S1.

### Dual-luciferase assays

Effector plasmids were transformed into the *A. tumefaciens* strain GV3101, and reporter plasmids were transformed into the strain GV3101-pSOUP. Overnight *A. tumefaciens* cultures were harvested by centrifugation at 5000 g for 15 min, resuspended in MES washing buffer to achieve an OD600 of 0.8. Equal parts of Agro expressing each effector and RK19 were mixed, and Agro expressing the reporter was added at 1/10 of the volume. This mixture was incubated at room temperature for 20 min to 2 h before infiltration into *N. benthamiana* leaves using a needleless syringe. The infiltrated tobacco plants were grown for 2 to 3 days under standard long-day conditions. Tissue samples were taken from the infiltrated area with an 8 mm biopsy skin punch (Mediware), immersed in 100 μL 1× lysis buffer (Dual-Luciferase Assay kit, Promega), and lysed in a 96-well plate using the flat side of a plastic pestle (Merck). The LUC and REN luminescence were generated as per kit instructions (Dual-Luciferase Assay kit, Promega) and detected using a spectrophotometer (Tecan Infinite 200 PRO).

### Molecular cloning

All pFAST effectors were cloned using LR reactions. For the reporters, *proSULTR1.1* and *proSULTR1.1* was amplified from *Arabidopsis thaliana* gDNA using the primers mentioned in this study. These 2 promoter fragments were each cloned into pGreenII0800 using Vazyme assembly via the PstI and ApaI restriction sites.

### Statistical analysis

Experimental values represent mean values and SE; *n* represents the number of independent samples. Significant differences between groups were analyzed by 1-way ANOVA or 2-way ANOVA followed by Tukey's post hoc test or *t* test for multiple comparisons in R (https://www.Rproject.org) and or JMP software *(*https://www.jmp.com/en/academic*)*.

## Supplementary Material

kiag444_Supplementary_Data

## Data Availability

The data underlying this article are available at DataPLANT (an NFDI inititative) under the project id 4239. https://git.nfdi4plants.org/ceplas/TGA_Basu26/-/tree/18ee1a1b483e7dcbfdf164c2147842fc6bf4c0e0/.

## Materials & correspondence

The author responsible for distribution of materials integral to the findings presented in this article in accordance with the policy described in the Instructions for Authors (https://academic.oup.com/plcell/pages/General-Instructions) is: Daniela Ristova (dristova@uni-koeln.de).
