# Supplementary material for: The primary nitrate response TGA1 and TGA4 transcription factors are negative regulators of sulfate uptake and metabolism
Source: Plant Physiol. 2026 Jul 6;201(4):kiag444. doi: 10.1093/plphys/kiag444 (PMC13524079; doi:10.1093/plphys/kiag444)
Supplement: Supplementary file 1 [file kiag444_supplementary_data.zip › TGApaper_SuppFig_revision.pdf]

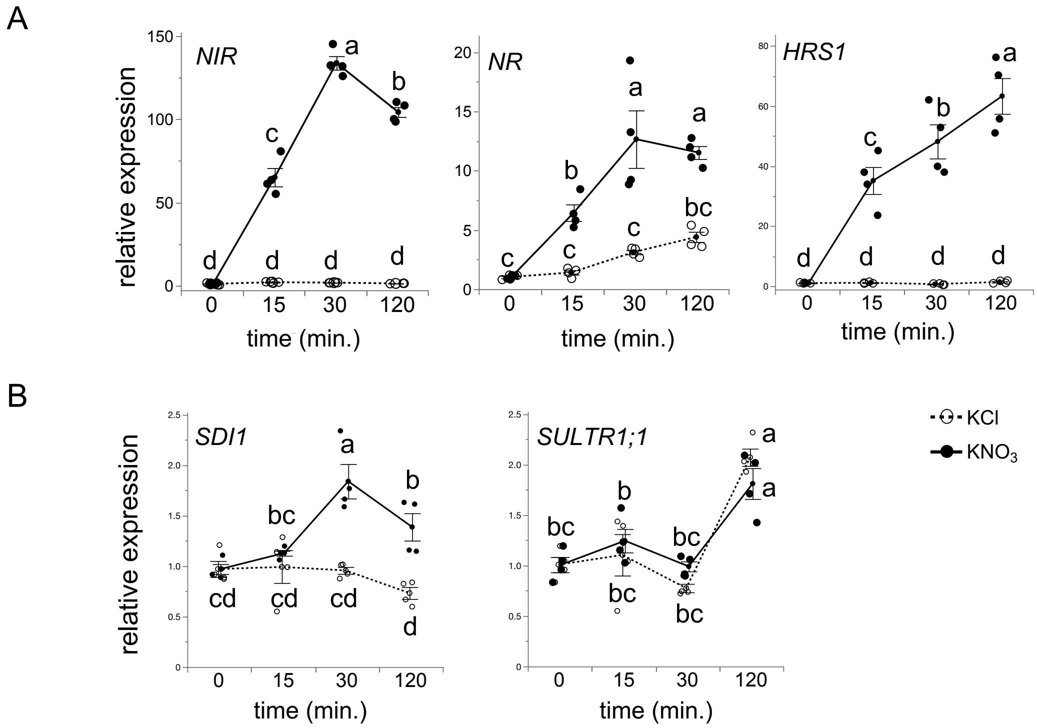

Supplementary Figure S1. Nitrate provision effect on gene expression. A) Nitrate responsive sentinels: dynamic response of mRNA for *NIR (NITRITE REDUCTASE), NR* (*NITRATE REDUCTASE*) and *HRS1* (*HYPERSENSITIVITY TO LOW PI-ELICITED PRIMARY ROOT SHORTENING 1*). **B)** Sulfate starvation responsive genes: *SDI1 (SULPHUR DEFICIENCY-INDUCED 1), and SULTR1;1 (SULFATE TRANSPORTER 1;1).* Statistical analysis was performed using two-way ANOVA followed by LS Means *t-test* for multiple comparisons for treatment and time, values are means ± s.e.m. (n=4). Different letters indicate significant differences (P<0.05) between the time and the treatments. 14-day-old wild-type Col-0 seedlings were grown hydroponically, and treated with 1 mM KNO_3 or 1 mM KCl (as mock treatment) for 15, 30 or 120 minutes.

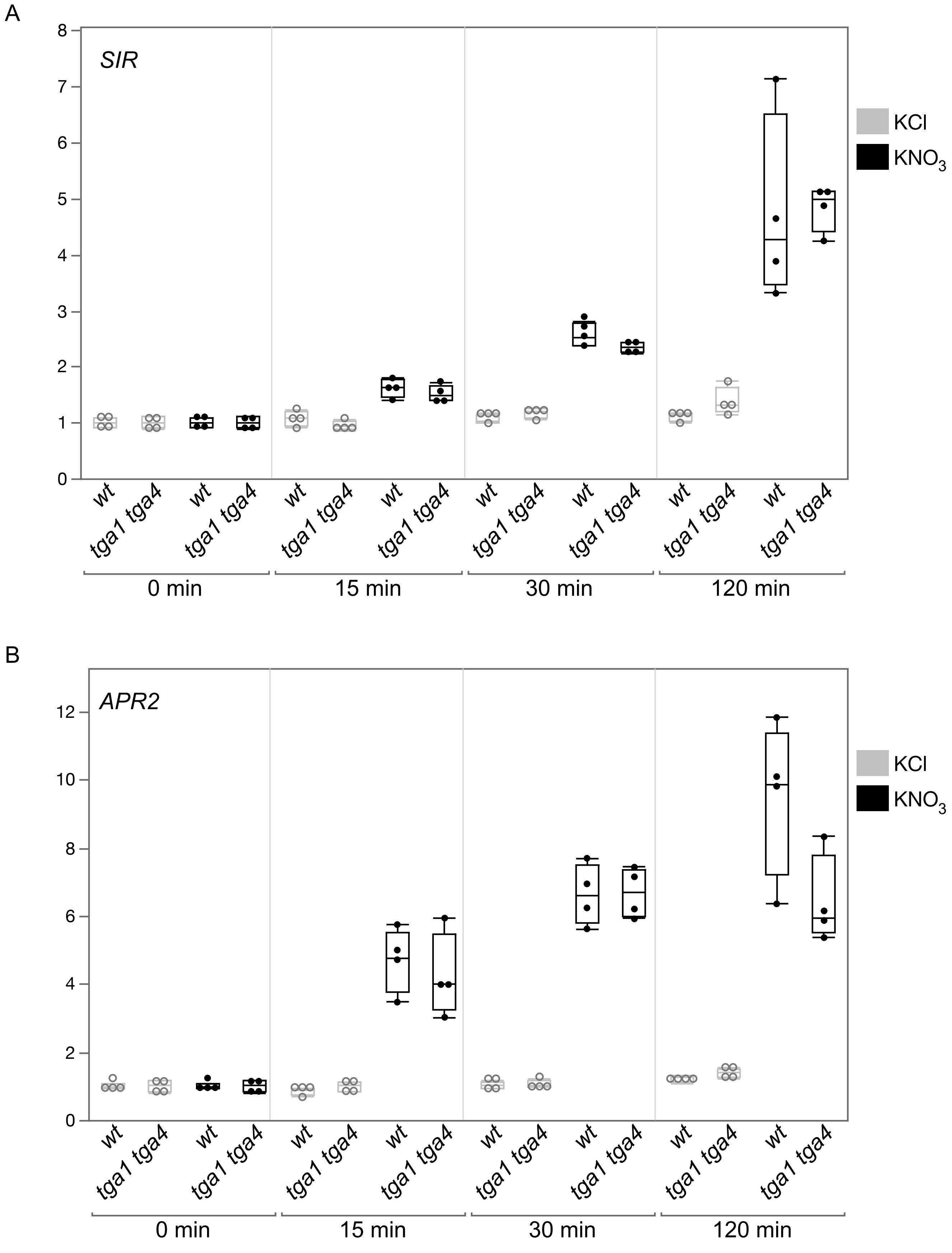

Supplementary Figure S2. Relative expression of sulfate assimilation genes. (A) Dynamic response of mRNA for *SIR* transcript levels and **(B)** Dynamic response of mRNA for *APR2* transcript levels, in wild-type Col-0 (*wt*), and the double mutant *tga1 tga4* in roots of 14-day-old seedlings grown hydroponically treated with 1mM KNO_3 or 1mM KCl (as mock treatment). Values are means ± s.e.m. (n=4). Statistical analysis was performed using two-way ANOVA followed by LS Means t-test for multiple comparisons for treatment and genotype for each time-point. No significant differences ($P<0.05$) was observed between the genotypes in the same treatments for each time-point.

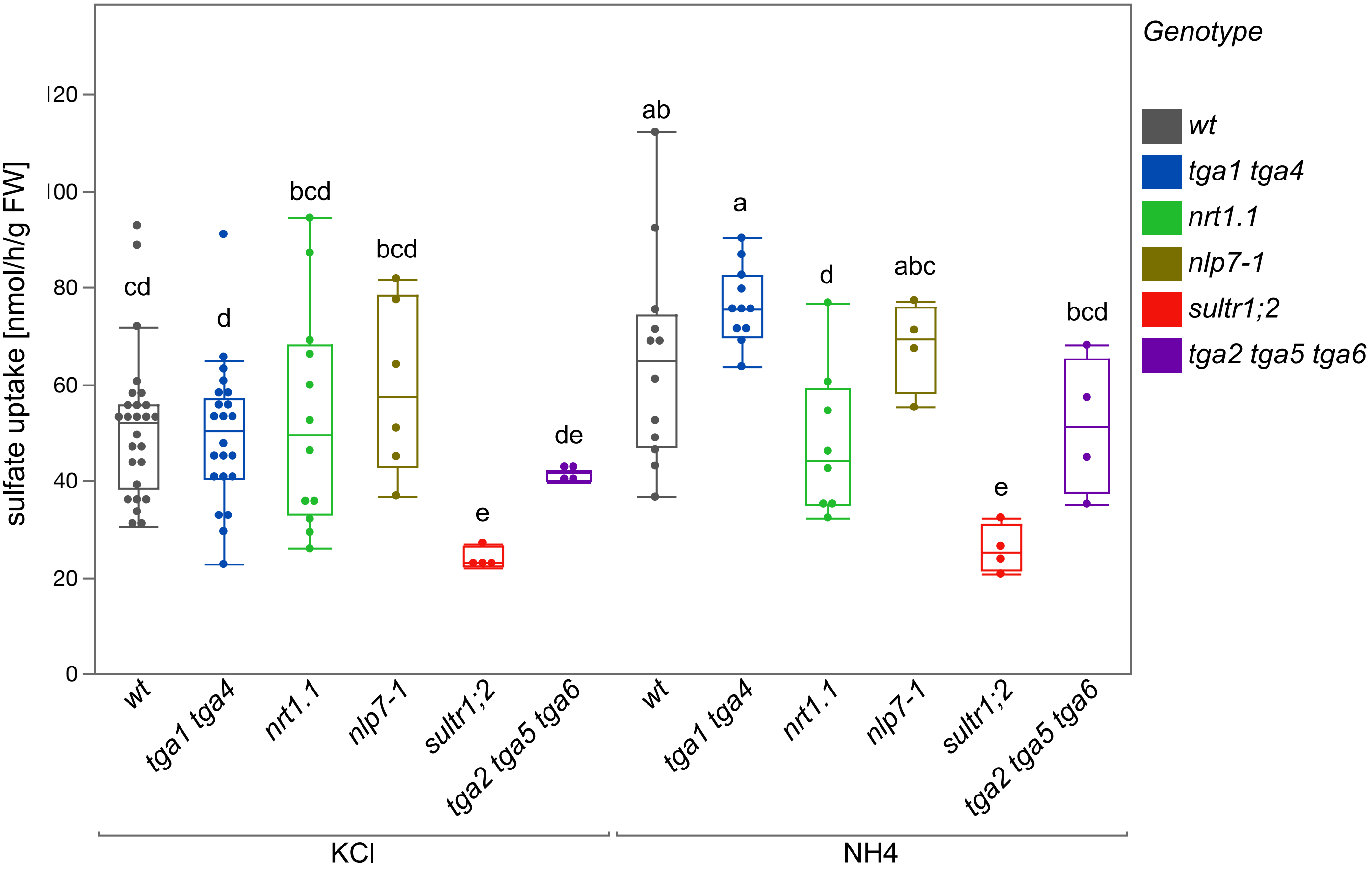

Supplementary Figure S3. Sulfate uptake increase by ammonium is not dependent on TGA1 and TGA4. Sulfate uptake (µmol/g FW) in *wt*, *tga1 tga4*, *tga2 tga5 tga6*, *nrt1.1*, *nlp7-1* and *sultr1;2* upon ammonium provision at 2 h. Arabidopsis plants (25 seedlings) were grown on a nylon net in sterile hydroculture for 13 days full-media, followed by 24 h of N starvation, and after incubated with control treatment (KCl) or ammonium chloride (NH_4Cl). For sulfate uptake, additional incubation with [^{35}S] sulfate for 30 min. was performed, and shoots and roots were harvested separately and extracted with 0.1 M HCl for radioactivity quantification. Statistical analysis was performed using two-way ANOVA followed by LS Means *t-test* for multiple comparisons for treatment and genotype, values are means ± s.e.m. (n=4-20). Different letters indicate significant differences (P<0.05) between the genotypes and the treatments.

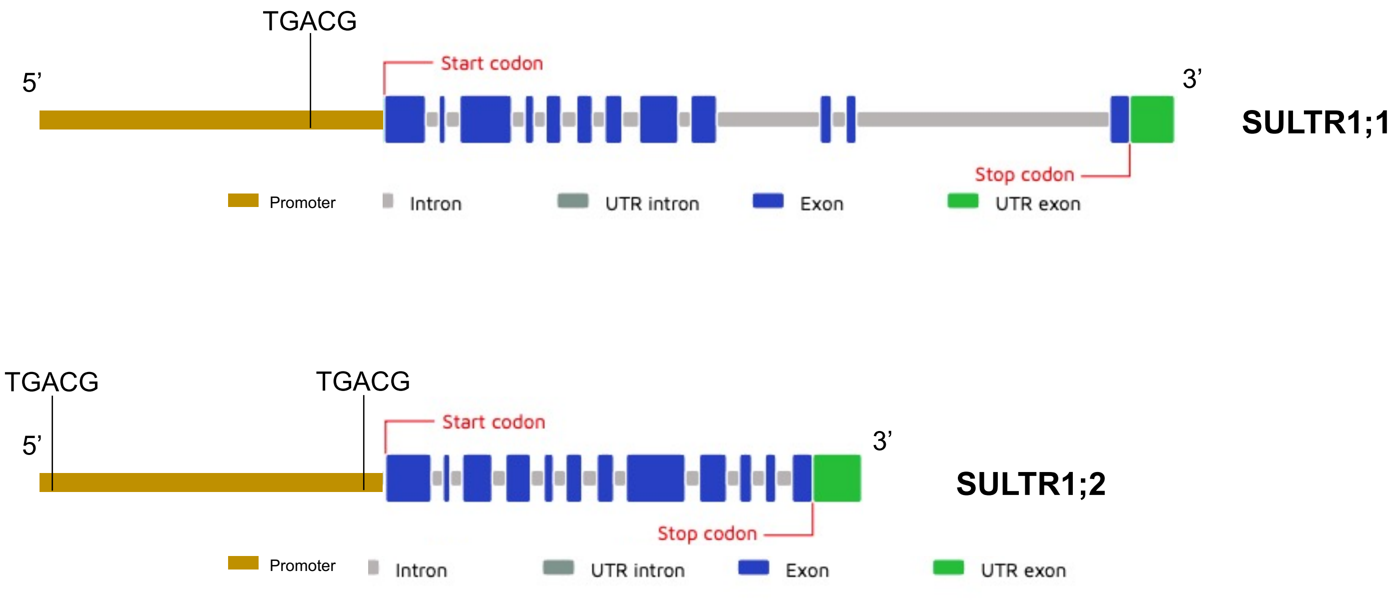

Supplementary Figure S4. Schematic representation of SULTR1;1 and SULTR1;2 genes and the TGA binding site in the 2kb promoter. TGA binding site is present in the promoters of *SULTR1;1* at 403 bp from transcription start site (TSS), while two TGA binding sites are present in the promoters of *SULTR1;2* at 37 bp, and 1980 bp from TSS, in the 2 kb upstream region. Gene models are created in PLAZA 5.1.

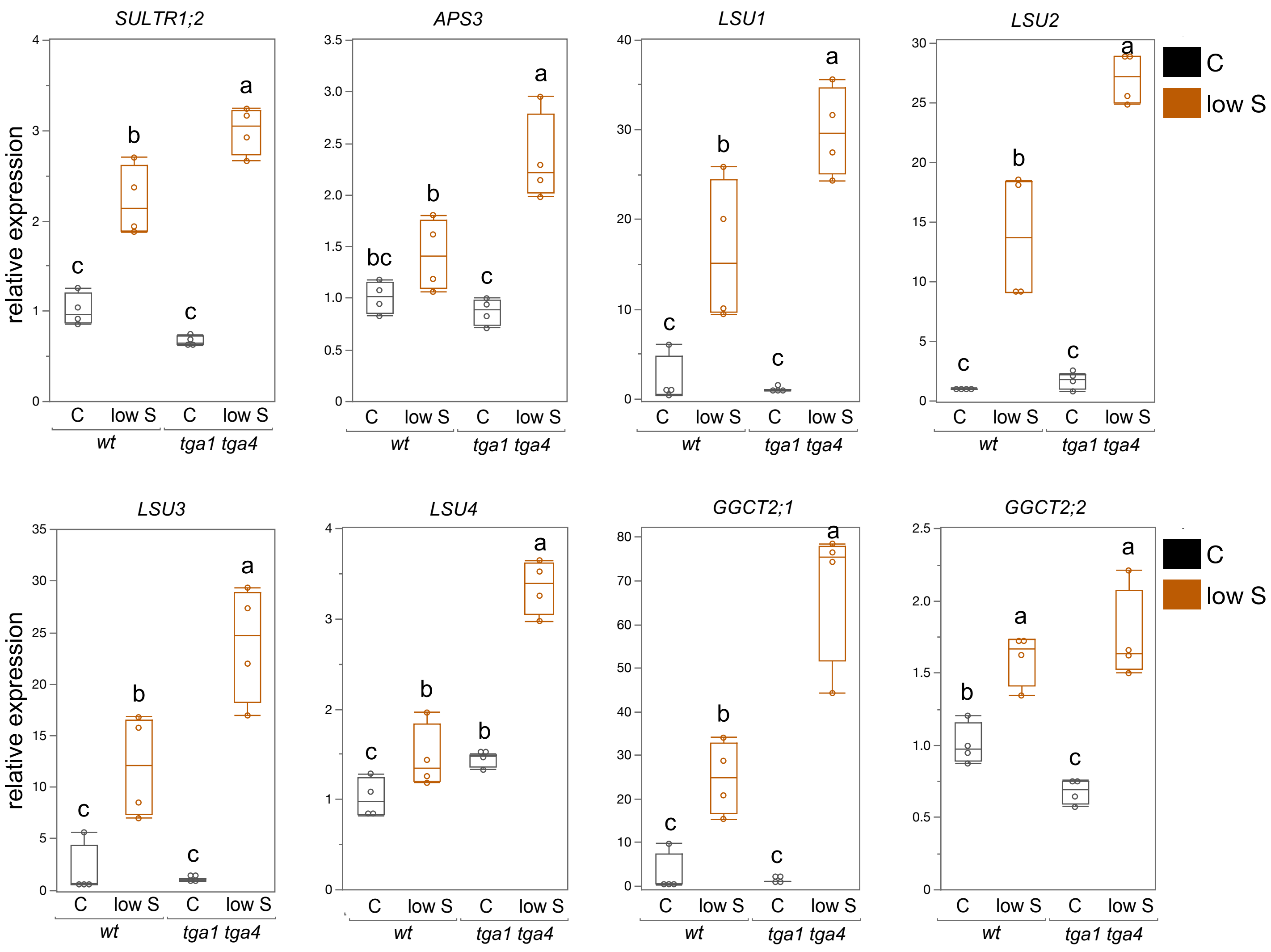

Supplementary Figure S5. Clade I TGA transcription factors (TGA1 and TGA4) are negative regulators of sulfate transport, assimilation and homeostasis regulation under S-deficiency. Quantitative reverse transcription PCR analysis of eight genes in wild-type Col-0 (*wt*), and *tga1 tga4* roots, under control (C) and S-deficiency (low S). Plants were grown vertically on plates for 18 d on either 0.75mM $SO4^{2-}$ (C) or 0.015mM $SO4^{2-}$ (low S) on vertical plates. Relative gene expression was determined via reverse transcription quantitative PCR (RT-qPCR) normalized to two housekeeping genes. Statistical analysis was performed using two-way ANOVA followed by Tukey's post hoc test for multiple comparisons for treatment and genotype. Different letters indicate significant differences (P<0.05) between the genotypes and the treatments. Values are means ± s.e.m. (n=4).

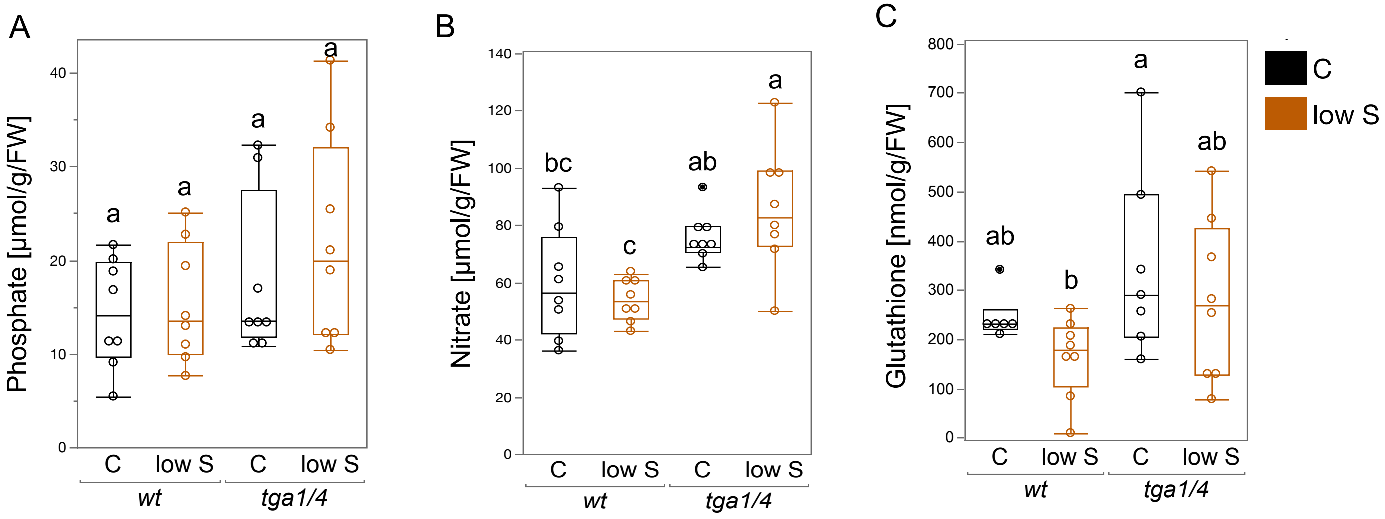

Supplementary Figure S6. Content of phosphate, nitrate and glutathione in clade I TGA transcription factors mutant (*tga1 tga4*) under long-term S-deficiency on pots. (A) Phosphate anions in shoots (µmol/g FW). **(B)** Nitrate anions in shoots (µmol/g FW). **(C)** Glutathione levels (µmol/g FW) in shoots. Wild-type (*wt*), and the double mutant *tga1 tga4* (*tga1/4*) were grown under long-term full-media (C), or S-deficient media (low S) in the green house. Pots with ratio 9:1 (sand:soil) were used and full-media or media lacking sulfate was added twice weekly. For metabolic phenotypes, leaf 7 and 8 were harvested 35 days after sowing, and used for anions, thiols, and glucosinolate isolation. Statistical analysis was performed using two-way ANOVA followed by LS Means *t-test* for multiple comparisons for treatment and genotype, values are means ± s.e.m. (n=4-8). Different letters indicate significant differences (*P*<0.05) between the genotypes and the treatments.

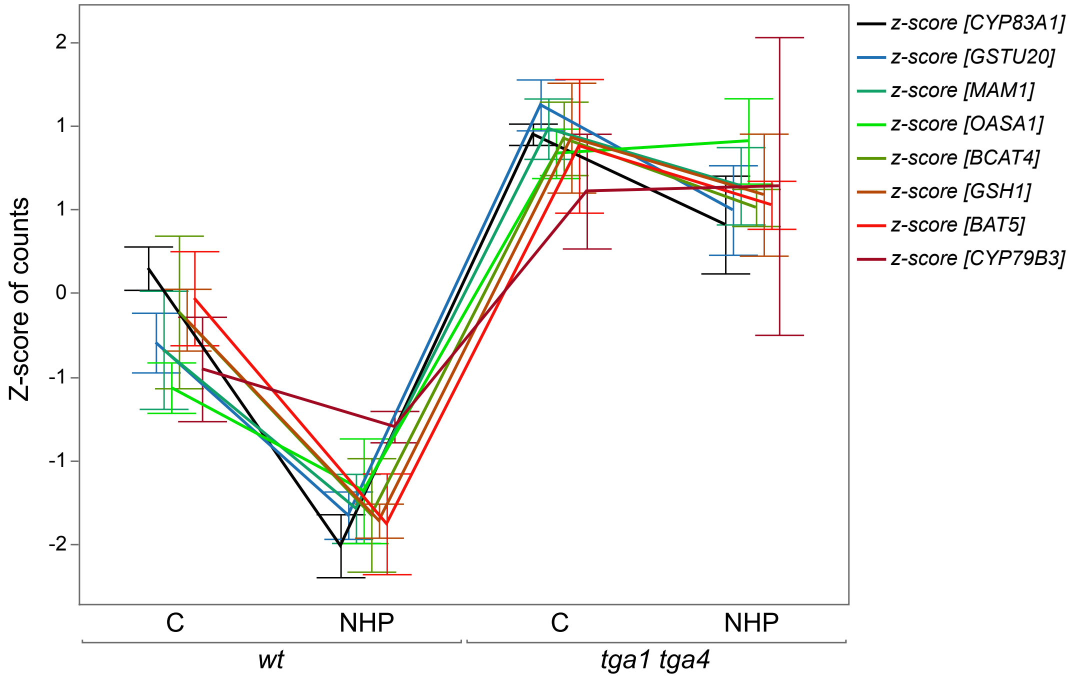

Supplementary Figure S7. Upregulated expression of glucosinolates genes in the *tga1 tga4* mutant under control (C) and N-hydroxypipecolic acid (NHP). Normalized counts were obtained from previous published study (Yildiz et al., 2023). By re-analyzing this dataset and implementing DESeq2 analysis with interaction term, we revealed that *tga1 tga4* genotype had enriched GO terms of "glucosinolate metabolic process". Here we show 8 genes that follow similar pattern of upregulation in the double *tga1 tga4* mutant in both conditions.

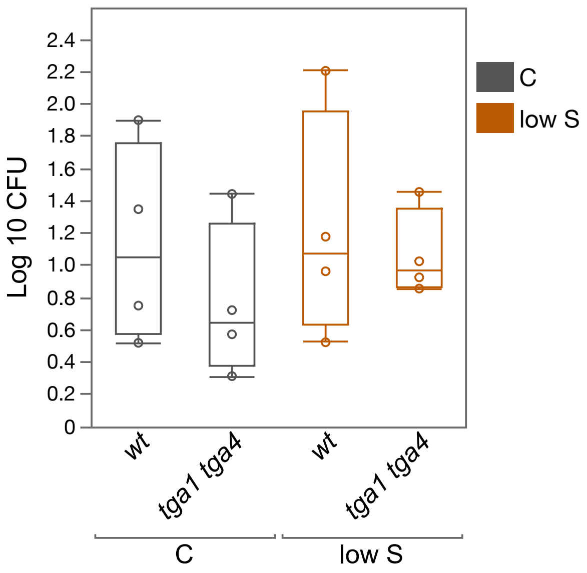

Supplementary Figure S8. Bacterial titer showed comparable levels in both conditions and genotypes after infection with BG (*B. glumae* PG1). Arabidopsis plants (25 seedlings) were grown on a nylon net in hydroculture for 10 days in 0.75mM $SO4^{2-}$ (C) or 0.015mM $SO4^{2-}$ (low S), followed by inoculation with BG bacteria. After 3 days shoots and roots were harvested, and DNA was isolated from the roots and subjected to qPCR with primers against *B. glumae* PG1 and the Arabidopsis AT4G26410 gene as a control. Using a previously established calibration between Ct values, OD, and CFU, the qPCR data were expressed as CFU (Ross and Somssich, 2016), presented as means ±SE from four biological replicates.

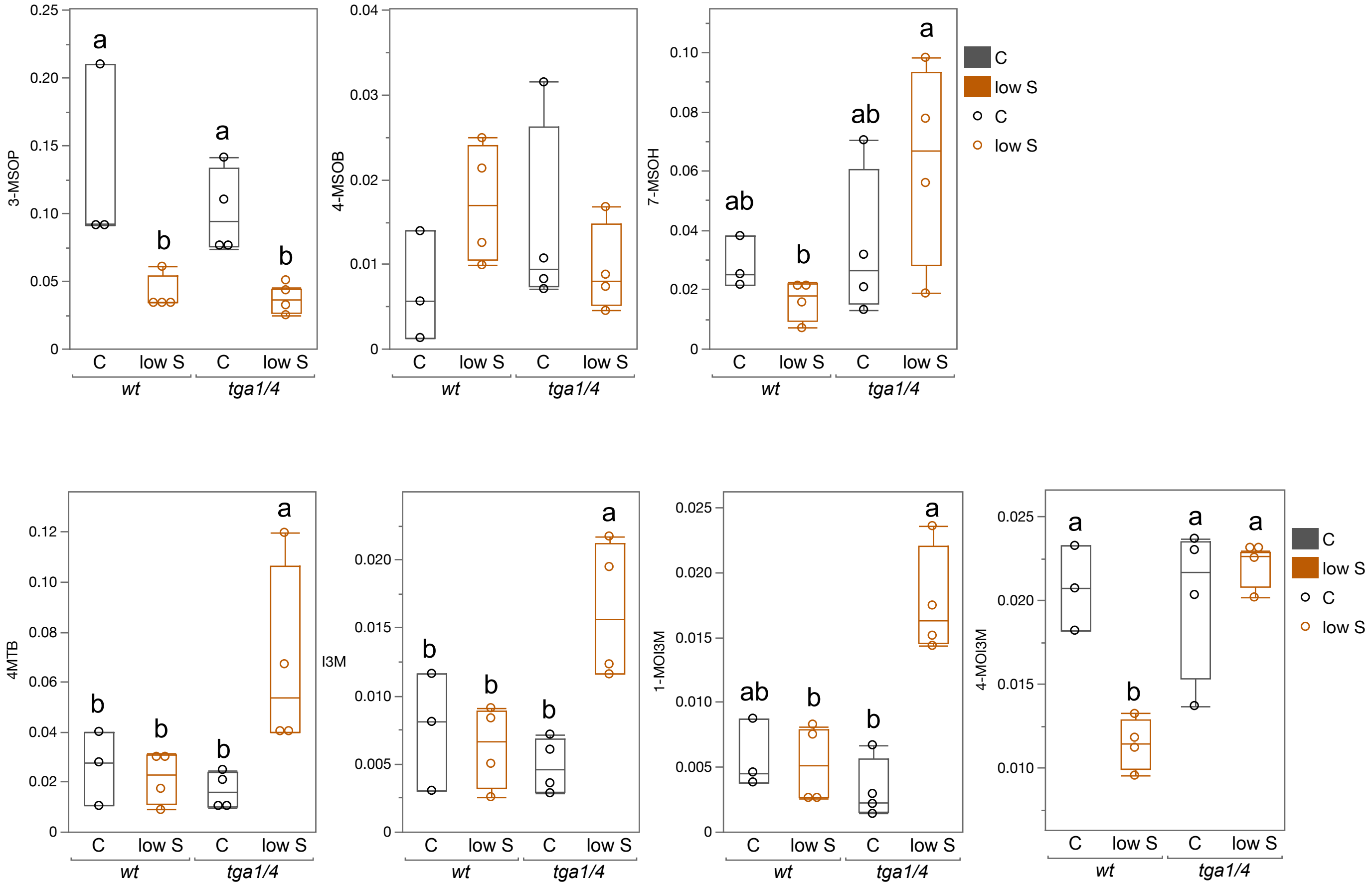

Supplementary Figure S9. Quantification of glucosinolates metabolites *tga1 tga4* mutant infected with BG (*B. glumae* PG1). Aliphatic glucosinolates: 3-MSOP (3-Methylsulfinylpropyl), 4-MSOB (4-methylsulfinylbutyl), 7MSOH (7-methylsulfinylheptyl), and indolic GSLs 4-MTB (4-methylthiobutyl), I3M (indol-3-ylmethyl), 1-MOIM (1-methoxyindol-3-ylmethyl), 4-MOIM (4-methoxyindol-3-ylmethyl) in wild-type Col-0 (*wt*), and *tga1 tga4 (tga1/4)* genotypes. Arabidopsis plants (25 seedlings) were grown on a nylon net in hydroculture for 10 days in 0.75mM $SO4^{2-}$ (C) or 0.015mM $SO4^{2-}$ (low S), followed by inoculation with BG bacteria. After 3 days shoots and roots were harvested. Relative gene expression was determined via reverse transcription quantitative PCR (RT-qPCR) normalized to two housekeeping genes. Statistical analysis was performed using two-way ANOVA followed by LS Means *t-test* for multiple comparisons for treatment and genotype, values are means ± s.e.m. (n=3-4). Different letters indicate significant differences (P<0.05) between the genotypes and the treatments.

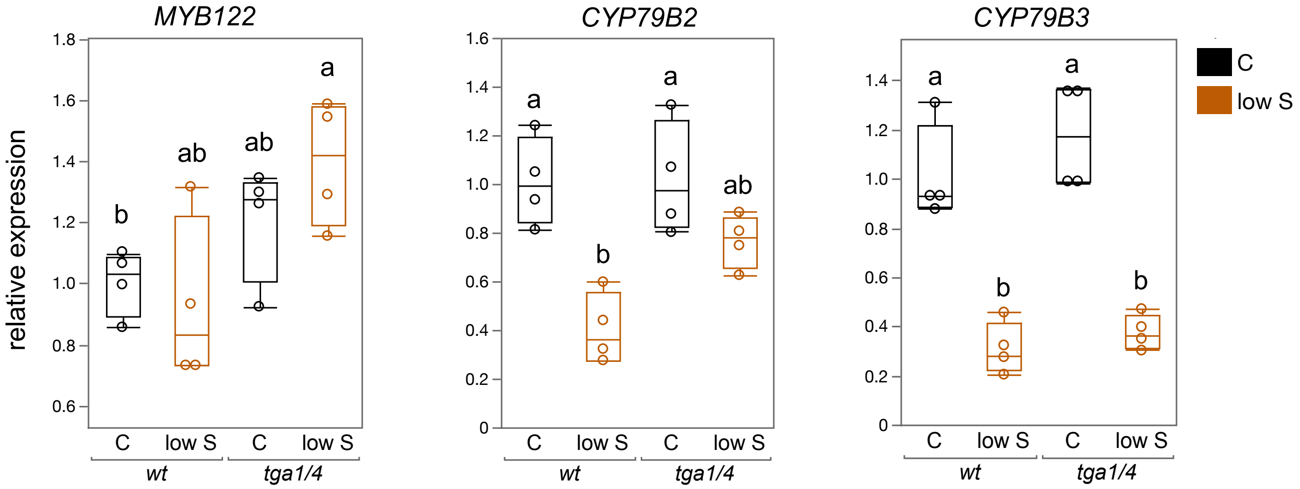

Supplementary Figure S10. Quantitative reverse transcription PCR analysis of indolic GLSs genes in wild-type Col-0 (*wt*), and *tga1 tga4 (tga1/4)* roots, under control (C) and S-deficiency (low S). Arabidopsis plants (25 seedlings) were grown on a nylon net in hydroculture for 10 days in 0.75mM $SO4^{2-}$ (C) or 0.015mM $SO4^{2-}$ (low S), followed by inoculation with BG bacteria. After 3 days shoots and roots were harvested. Relative gene expression was determined via reverse transcription quantitative PCR (RT-qPCR) normalized to two housekeeping genes. Statistical analysis was performed using two-way ANOVA followed by LS Means *t-test* for multiple comparisons for treatment and genotype, values are means ± s.e.m. (n=3-4). Different letters indicate significant differences (P<0.05) between the genotypes and the treatments.

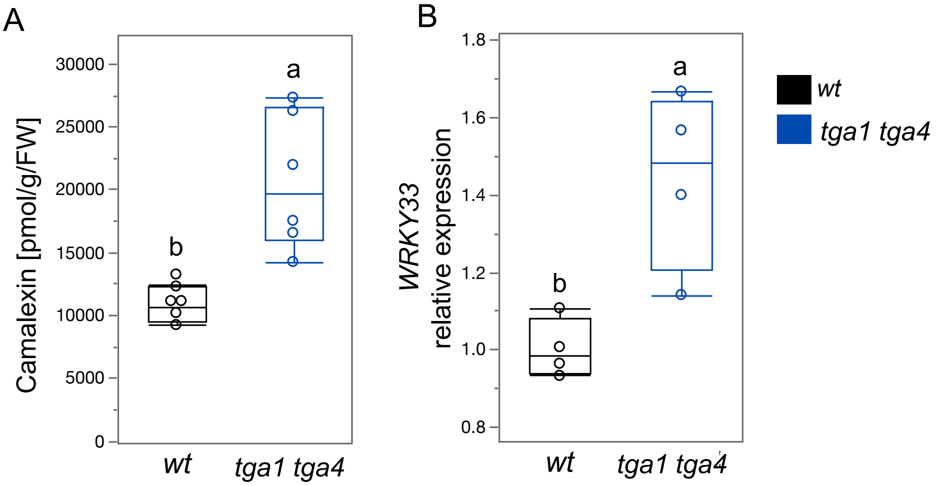

Figure S11. Camalexin profiles and expression analysis in double mutant *tga1 tga4* after long-term S-deficiency infected with BG. (A) Camalexin in shoots after infection with the pathogenic bacteria BG (*B. glumae* PG1). **(B)** Quantitative reverse transcription PCR analysis of *WRKY33* that directly regulates camalexin biosynthesis, in wild-type Col-0 (*wt*), and *tga1 tga4* roots. Arabidopsis plants (25 seedlings) were grown on a nylon net in hydroculture for 10 days in half-MS-media, followed by inoculation with BG bacteria. After 3 days shoots and roots were harvested. Relative gene expression was determined via reverse transcription quantitative PCR (RT-qPCR) normalized to two housekeeping genes. Statistical analysis was performed using one-way ANOVA followed by LS Means *t-test* for multiple comparisons for treatment and genotype, values are means ± s.e.m. (n=4-6). Different letters indicate significant differences (P<0.05) between the genotypes.
